# XdfA, a novel membrane-associated DedA family protein of *Xanthomonas campestris*, is required for optimum virulence, maintenance of magnesium, and membrane homeostasis

**DOI:** 10.1128/mbio.01361-23

**Published:** 2023-07-27

**Authors:** Yasobanta Padhi, Subhadeep Chatterjee

**Affiliations:** 1 Laboratory of Plant-Microbe Interactions, Centre for DNA Fingerprinting and Diagnostics, Hyderabad, Telangana, India; 2 Graduate Studies, Manipal Academy of Higher Education, Udupi, Karnataka, India; University of California Berkeley, Berkeley, California, USA; University of California Berkeley, Berkeley, California, USA

**Keywords:** DedA family protein, membrane integrity, magnesium transporter, virulence

## Abstract

*Xanthomonas campestris* is an important member of the *Xanthomonas* group of phytopathogens that causes diseases in crucifers. In *X. campestris*, several virulence-associated functions, including some belonging to unknown predicted functions, have been implicated in the colonization and disease processes. However, the role of many of these unknown predicted proteins in *Xanthomonas*-host interaction and their exact physiological function is not clearly known. In this study, we identified a novel membrane-associated protein belonging to the DedA super family, XdfA, which is required for virulence in *X. campestris*. The DedA family of proteins are generally ubiquitous in bacteria; however, their function and actual physiological role are largely elusive. Characterization of ∆*xdfA* by homology modeling, membrane localization, and physiological studies indicated that XdfA is a membrane-associated protein that plays a role in the maintenance of membrane integrity. Furthermore, functional homology modeling analysis revealed that the XdfA exhibits structural similarity to a CorA-like magnesium transporter and is required for optimum growth under low magnesium ion concentration. We report for the first time that a putative DedA family of protein in *Xanthomonas* is required for optimum virulence and plays a role in the maintenance of membrane-associated functions and magnesium homeostasis.

**IMPORTANCE:**

Bacterial DedA family proteins are involved in a range of cellular processes such as ion transport, signal transduction, and cell division. Here, we have discussed about a novel DedA family protein XdfA in *Xanthomonas campestris* pv. *campestris* that has a role in membrane homeostasis, magnesium transport, and virulence. Understanding membrane and magnesium homeostasis will aid in our comprehension of bacterial physiology and eventually will help us devise effective antimicrobial strategies to safeguard horticulturally and agriculturally important crop plants.

## INTRODUCTION

The *Xanthomonas* group of phytopathogens is known to cause many devastating diseases in agriculturally and horticulturally important crops, resulting in high economic loss. *Xanthomonas campestris* pv. *campestris* (*Xcc 8004*) causes black rot disease in cruciferous plants. This pathogen enters through hydathodes, the natural openings present on the leaf surface of plant hosts, to cause V-shaped necrosis on the leaves after successful establishment (1 - 3). *Xanthomonas* employ different secretory systems, effector molecules, extracellular enzymes for host specificity, and disease manifestation. Extracellular polysaccharide (EPS), secreted by *Xcc*, aids in interaction with the host as well as confers protection against hostile environments. Lipopolysaccharide (LPS), being an essential component of the outer membrane in Gram-negative bacteria, acts as a modulator of plant defense response in *Xanthomonas* (4 - 7). During the infection, many pathogenicity-associated bacterial genes work in coordination to exhibit various virulence-related functions for successful colonization of plants. Diffusible signaling factor-mediated cell−cell response or quorum sensing signaling is crucial for the bacteria to establish communication for a coherent response (8, 9). In addition to that, sequestration and maintenance of metal ion concentration play a major role in bacterial viability, host modulation, and successful invasion (10, 11).

In order to investigate other alternate virulence-associated functions in *Xcc*, we had earlier performed a genetic screen to identify mutants that are altered in virulence (12). Four mutants were identified that had transposon insertions in a DedA family of protein (XC_2523; named xdfA; Xanthomonas DedA family protein A), which had a significant virulence deficiency in cabbage in comparison to wild-type strain (*Xcc* 8004).

The highly conserved protein family DedA belongs to the “soluble N-ethylmaleimide-sensitive factor attachment protein receptors (SNARE-associated PF09335)” (13) family of proteins (PFAM 34.0). Although DedA family proteins are ubiquitous in several Gram-negative bacteria, their actual physiological role and cellular function are still poorly understood (14).

The DedA family proteins typically contain four to six transmembrane helices ranging from 200 to 250 amino acids. *Escherichia coli* has eight paralogs of DedA family proteins, out of which YqjA and YghB are widely studied. Mutants of these genes are reported to cause cell division defects, sensitivity to high temperatures, modified membrane lipid composition, increased envelope-related stress response, and impairment of proton motive force (14 - 16). In animal pathogenic bacteria such as *Klebsiella pneumoniae, Galleria mellonella* (a disease in wax moths), and *Burkholderia* sp., it has been shown that the DedA family protein is required for optimum virulence (17, 18). However, except for a single report on the rice bacterial panicle blight causing bacteria *Burkholderia glumae* (19), the role of this important family of proteins is poorly understood in phytopathogenic bacteria, and their specific role in cellular function is largely elusive.

In this study, we have elucidated the role of XdfA (a novel DedA family protein) in virulence-associated functions. We have demonstrated that *xdfA* is required for virulence-associated functions such as biofilm formation and EPS, and growth under low magnesium conditions.

## RESULTS

### XdfA is conserved across the members of order *Xanthomonadales* and other bacterial plant pathogens

**Fig 1:**
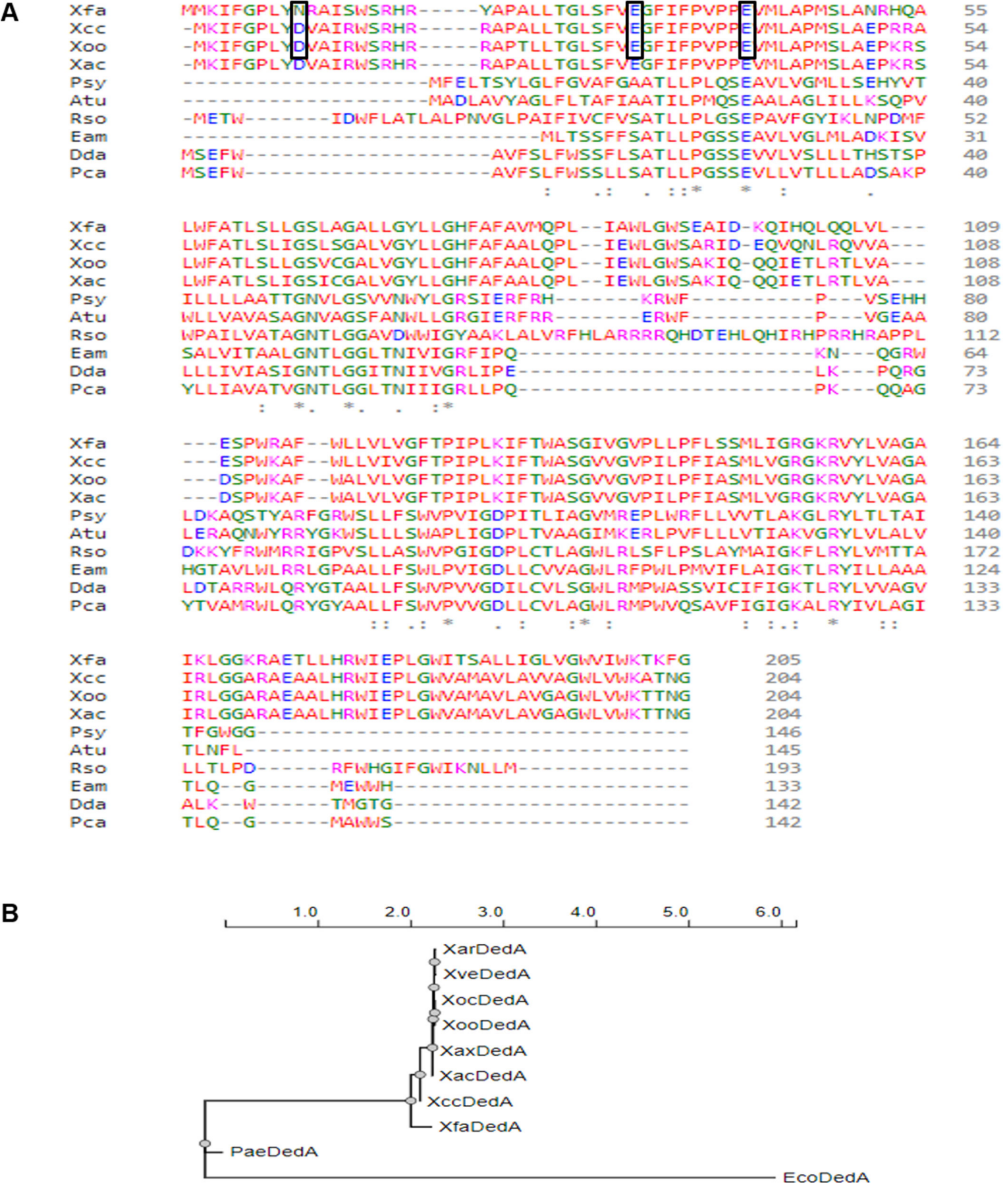
DedA sequence homology to deduce evolutionary ancestry. (A) Multiple sequence alignment of DedA family proteins from *Xylella fastidiosa* (Xfa), *Xanthomonas campestris* pv. *campestris* 8004 (Xcc), *Xanthomonas oryzae* pv. *oryzae* (Xoo), *Xanthomonas axonopodis* pv. *citri* (Xac), *Pseudomonas syringae* (Psy), *Agrobacterium tumefaciens* (Atu), *Ralstonia solanacearum* (Rso), *Erwinia amylovora* (Eam), *Dickeya dadantii* (Dda), and *Pectobacterium carotovorum* (Pca). The symbol ”*” indicates identical amino acids, ”:” indicates highly conserved, “.” indicates less conserved, and rectangular boxes indicate conserved negatively charged amino acids signifying putative metal-binding sites. (B) Phylogenetic dendrogram showing evolutionary interrelationship of *Escherichia coli* (Eco), *Pseudomonas aeruginosa* (Pae), *Xylella fastidiosa* (Xfa), *Xanthomonas campestris* pv. *campestris 8004* (*Xcc*), *Xanthomonas vesicatoria* (Xve), *Xanthomonas arboricola* (Xar), *X. axonopodis* (Xax), *Xanthomonas citri* pv. *citri* (Xac), *X. oryzae* pv. *oryzae* (Xoo), and *X. oryzae* pv. *oryzicola* (Xoc).

Using forward genetic screening, we have isolated three independent transposon induced mutants that were deficient in virulence with insertions at amino acid positions 17, 103, and 132 of the gene locus XC_2523, which belongs to the DedA family of proteins (Fig. S1; Table S1). The DedA family of proteins are highly conserved and belong to the SNARE-associated family of proteins (18). *E. coli* has eight paralogs of this family which are individually non-essential (20). We found only one copy of this gene in case of *Xcc* 8004, which we have denoted as *xdfA*. To understand the evolutionary conservation of XdfA throughout plant pathogens, we have done a multiple sequence alignment study using the online platform, ClustalOmega. The amino acid sequences of XdfA orthologs from three *Xanthomonas* species, their close relative *Xylella fastidiosa*, and a few other prominent plant pathogens like *Pseudomonas syringae, Agrobacterium tumefaciens*, *Ralstonia solanacearum*, *Erwinia amylovora*, *Dickeya dadantii*, and *Pectobacterium carotovorum* were aligned. All these proteins are 200 to 250 amino acids long and have a conserved glutamate site (Fig. 1A). In order to delineate conserved homologs of XdfA across these species, phylogenetic analysis was performed using the NCBI database, and a dendrogram was constructed using the PRESTO method (https://ngphylogeny.fr) after amino acid sequence alignment with ClustalOmega. Phylogenetic analysis suggested that, XdfA has more than 90% identity with the DedA family proteins of other members of the *Xanthomonas* genus as well as 77% identity with the DedA family protein from *Xylella fastidiosa*. However, its identity with *Pseudomonas aeruginosa* stands at nearly 34%. Overall, the result suggests that XdfA is conserved among the pathogens that belong to the family *Xanthomonadales* and is distantly related to its orthologs in other Gram-negative bacteria, *P. aeruginosa* and *E. coli* (Fig. 1B).

### ∆*xdfA* exhibits significant reduction in virulence as well as *in planta* migration

**Fig 2:**
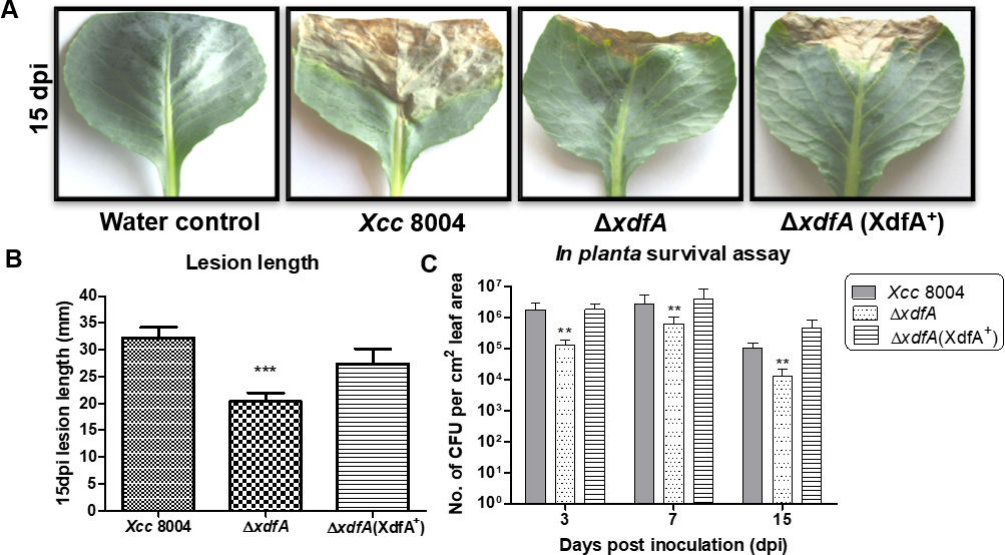
*In planta* survival and virulence efficiency in the absence of *xdfA*. (A) Representative images of infected cabbage leaves with different *Xcc* strains, showing symptoms as a lesion, 15 days post-clip inoculation. (B) Quantification of lesion length at 15 days post-inoculation. Data are shown as mean ± standard deviation (SD) (*n* = 25). (C) Temporal dynamics of bacterial population size determined *in planta* at designated post-inoculation days. Data are shown as mean ± SD (*n* = 3) and statistical significance by paired Student’s *t* test (**P < 0.01, ***P < 0.001).

To study the role of *xdfA* in the virulence-associated functions of *Xcc*, an in-frame marker-free deletion was created in the *xdfA* locus (XC_2523) to get ∆*xdfA*. Forty-five days old cabbage plants were clip-inoculated with wild type as well as mutant ∆*xdfA* and chromosomally complemented ∆*xdfA* (XdfA^+^) strains of *Xcc. Xcc* siderophore utilization mutant ∆*xsuA* was used as a negative control (data not shown). Observation of lesion length was taken 14 days after clip inoculation. ∆*xdfA* exhibited nearly 50% reduction in lesion length (Fig. 2A)*,* which is the prominent phenotype of black rot caused by *Xcc* in cruciferous plants. The reduction in lesion length is almost twofold in comparison to wild-type *Xcc* (Fig. 2B).

To check the growth and migration of different strains of *Xcc* inside the cabbage plants, *in planta* migration and colony forming unit (CFU) experiments were performed. Cabbage leaves were detached 3 days after inoculation and surface sterilized. These were cut into 1 cm broad pieces with the help of sterile scissors and kept over a nutrient-rich peptone sucrose agar (PSA) plate incubated subsequently at 28°C. Observation of those leaves 3 days post-incubation revealed that ∆*xdfA* bacteria were unable to migrate more than 1 cm from the site of inoculation (Fig. S2), whereas wild-type *Xcc* colonies were oozed out 3 cm away from the site of inoculation. These findings suggest that XdfA is required for bacterial migration *in planta*, and that in the absence of XdfA, bacteria fail to migrate properly and cause disease in the host. Additionally, temporal dynamics of *in planta* survival of the bacteria were assessed by crushing the leaves after surface sterilization and growing them on PSA plates at early(3 days post infection [dpi])], middle (7 dpi), and late (15 dpi) infection stages. The results obtained clearly indicate a more than 10-fold decrease in the survival efficiency of ∆*xdfA* inside the cabbage host (Fig. 2C).

### ∆*xdfA* exhibits reduced biofilm formation and attachment in *Xcc* 8004

**Fig 3:**
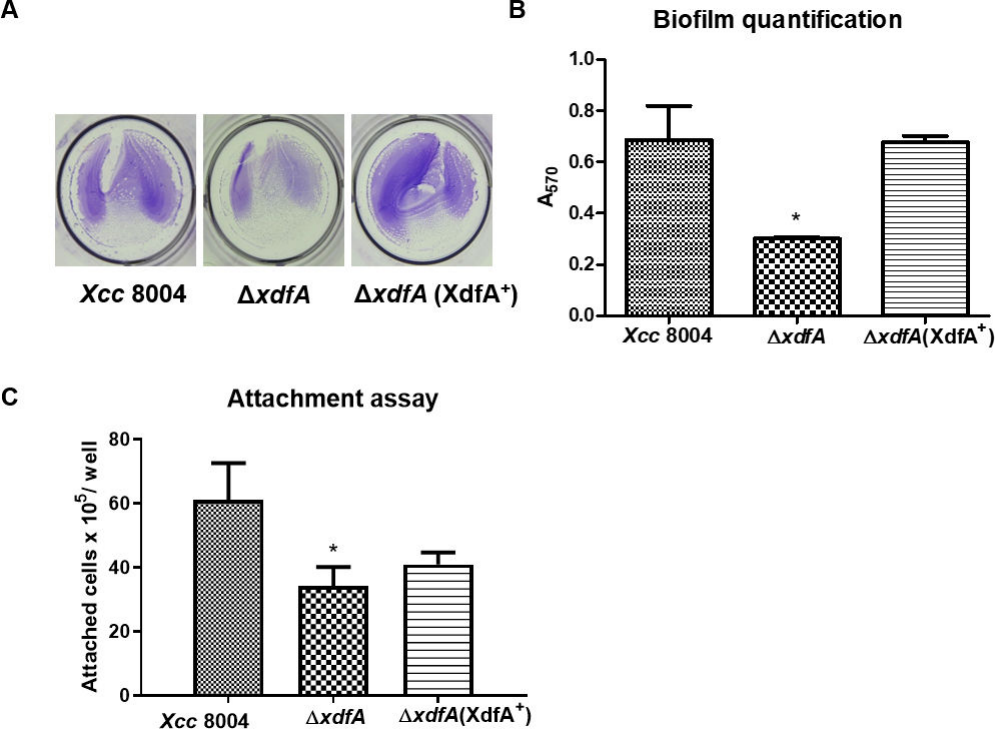
Effect of *xdfA* mutation on biofilm formation and surface attachment. (A) Representative image of bacterial biofilm formation of various *Xcc* strains after 16 h of incubation at 28°C and staining with 0.1% crystal violet. (B) Quantification of bacterial biofilm by staining with crystal violet and measuring absorbance at 570 nm post 90% ethanol wash. (C) CFU quantification of attached cells to the 12-well plate after 16 h of static incubation at 28°C and subsequent serial dilution before plating. Error bars represent ±SD of absorbance of crystal violet staining and CFU of attached cells obtained from three independent experiments. Statistical significance by paired Student’s *t* test (*P < 0.05).

Bacteria are known to form a consorted multicellular aggregate called biofilm on biotic as well as abiotic surfaces for successful invasion into hosts. To understand the role of XdfA in altering the biofilm structure, different strains of *Xcc* were grown in static conditions in 12-well polystyrene culture plates (8). The formation of biofilm biomass was assessed after 48 h of incubation using crystal violet to stain it and 90% ethanol to scrape and quantify it. It was observed that ∆*xdfA* is severely deficient in biofilm formation (Fig. 3A and B), whereas the chromosomal-complemented strain ∆*xdfA* (XdfA^+^) is as efficient as the wild-type *Xcc*.

In a similar fashion, the surface-adhered planktonic cell mass was washed with sterile water before serial dilution and plating to assess the bacterial count as CFUs on PSA plates. It was observed that ∆*xdfA* exhibited approximately two-fold reduced attachment in comparison to wild-type *Xcc* (Fig. 3C).

### ∆*xdfA* is deprived of producing EPS and phospholipids

**Fig 4:**
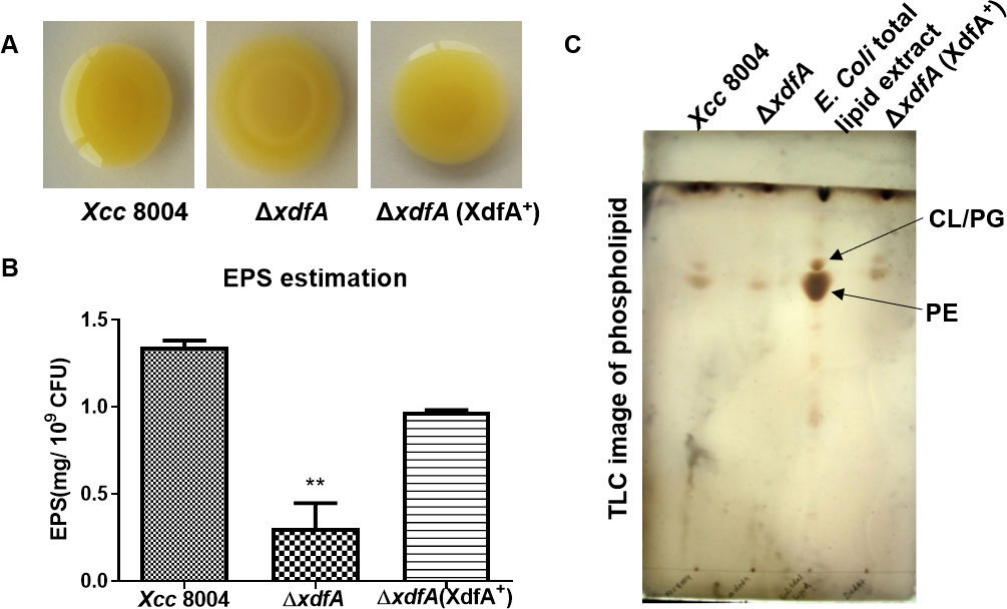
Profiling of EPS and phospholipids in the absence of *xdfA*. (A) Secreted extracellular polysaccharides (EPS) observed on PSA plates after 48 h of incubation at 28°C. (B) Quantification of EPS using phenol-sulfuric acid and measured at 490 nm. (C) Thin layer chromatography profiling of the phospholipids extracted from sodium acetate-treated optical density-normalized cultures of the wild-type *Xcc* 8004*,* ∆*xdfA,* and ∆*xdfA* (XdfA^+^) complement strains. *E. coli* total lipid extract (Sigma) was used as a standard. The expected spots for CL/PG and PE were marked with arrows (CL, cardiolipin; PG, phosphatidylglycerol; PE, phosphatidylethanolamine). These experiments were performed as three biological replicates. Data in (B) are shown as mean ± SD (*n* = 3) and statistical significance by paired Student’s *t* test (**P < 0.01).

The DedA family of proteins in *E. coli* have been reported to be essential for maintaining membrane homeostasis under alkaline and high osmotic conditions (15). To deduce their role in *Xanthomonas*, we have checked the growth of *Xcc* along with ∆*xdfA* and ∆*xdfA* (XdfA^+^) under altered pH and high osmotic conditions. All three strains were unable to grow in either extreme acidic (pH 2 to pH 5) or basic conditions (pH 10). However, among the three strains, there was no significant difference in the growth pattern at pH 6.0, pH 7.0, pH 7.4, pH 8.0, and pH 9.0 (Fig. S3). The growth pattern in the dilution spotting experiment on osmolytes like mannitol, sorbitol, and sucrose remained same for the three strains (Fig. S4). To check for any alteration in the growth pattern of these *Xcc* strains in the presence of phenolics and detergents, a dilution spotting assay was performed. Growth pattern in the presence of phenol, berberine chloride, and acetosyringone was similar, whereas growth of ∆*xdfA* was 10-fold less in the presence of rhein (Fig. S5). However, all the three strains were equally sensitive to tested surfactants SDS, Triton X-100, and Tween 20. To further characterize membrane-associated functions, we have examined the production of EPSs, LPSs, and glycerophospholipids. The EPS xanthan secreted by *Xcc* is one of the major factors in inciting disease in cruciferous host plants (21) . EPS-producing ability of various *Xcc* strains on nutrient-rich PSA plates revealed that the mutant of *xdfA* is severely compromised in its production and appears rather dry (Fig. 4A). The production of EPS in ∆*xdfA* was quantified to be approximately four times less in comparison to wild-type *Xcc*, in the phenol-sulfuric acid estimation (Fig. 4B). However, the chromosomal complemented strain ∆*xdfA* (XdfA^+^) was able to revert the EPS production to a large extent.

Mitigating envelope stress is crucial for successful colonization inside the host environment. LPS and glycerophospholipids, present in the outer membrane of Gram-negative bacteria, provide the pathogen with a firm barrier against hostile host-secreted molecules and immune responses (22, 23). To find out the probable role of outer envelope in maintaining homeostasis inside the host and to correlate it with reduced virulence and migration of ∆*xdfA* inside the host, we performed LPS visualization by silver nitrate staining followed by SDS-PAGE and phospholipid analysis by thin layer chromatography (TLC). The phospholipid profile of wild-type *Xcc*, ∆*xdfA,* and ∆*xdfA* (XdfA^+^) was checked in TLC along with commercially available *E. coli* total lipid extract as a control. As envisaged, the phospholipid profile of ∆*xdfA* was significantly altered compared to the wild type, and the phenotypes got restored by chromosomal complementation of the gene (Fig. 4C). But no significant change in the banding pattern of ‘O-antigen’ among the *Xanthomonas* strains was observed in the silver nitrate gel run after LPS isolation (Fig. S6).

We infiltrated wild-type *Xcc* 8004, ∆*xdfA*, and ∆*xdfA* (XdfA^+^) into tomato and benthi leaves in order to investigate the capacity of *Xcc* strains to elicit a hypersensitive response (HR) in non-host plants. Forty-eight hours after infiltration, different strains were able to induce HR. According to our observation, ∆*xdfA* was inducing increased HR in both the non-hosts (Fig. S7A and B). Type III secretion system (T3SS) and their effectors from bacterial pathogens are responsible for eliciting hypersensitive response in non-hosts (24). Hence, we have checked *in planta* promoter expression of three T3SS-related genes, *avrXccE*, *hrpX,* and *hrcU,* by making β-glucuronidase (GUS)-reporter fusion. The expression of *hrcU* promoter was significantly less in the ∆*xdfA* background in comparison to the *Xcc* background (Fig. S7C). These results apprehend the role of *xdfA* in the T3SS pathway.

### XdfA is getting localized into the inner membrane

**Fig 5:**
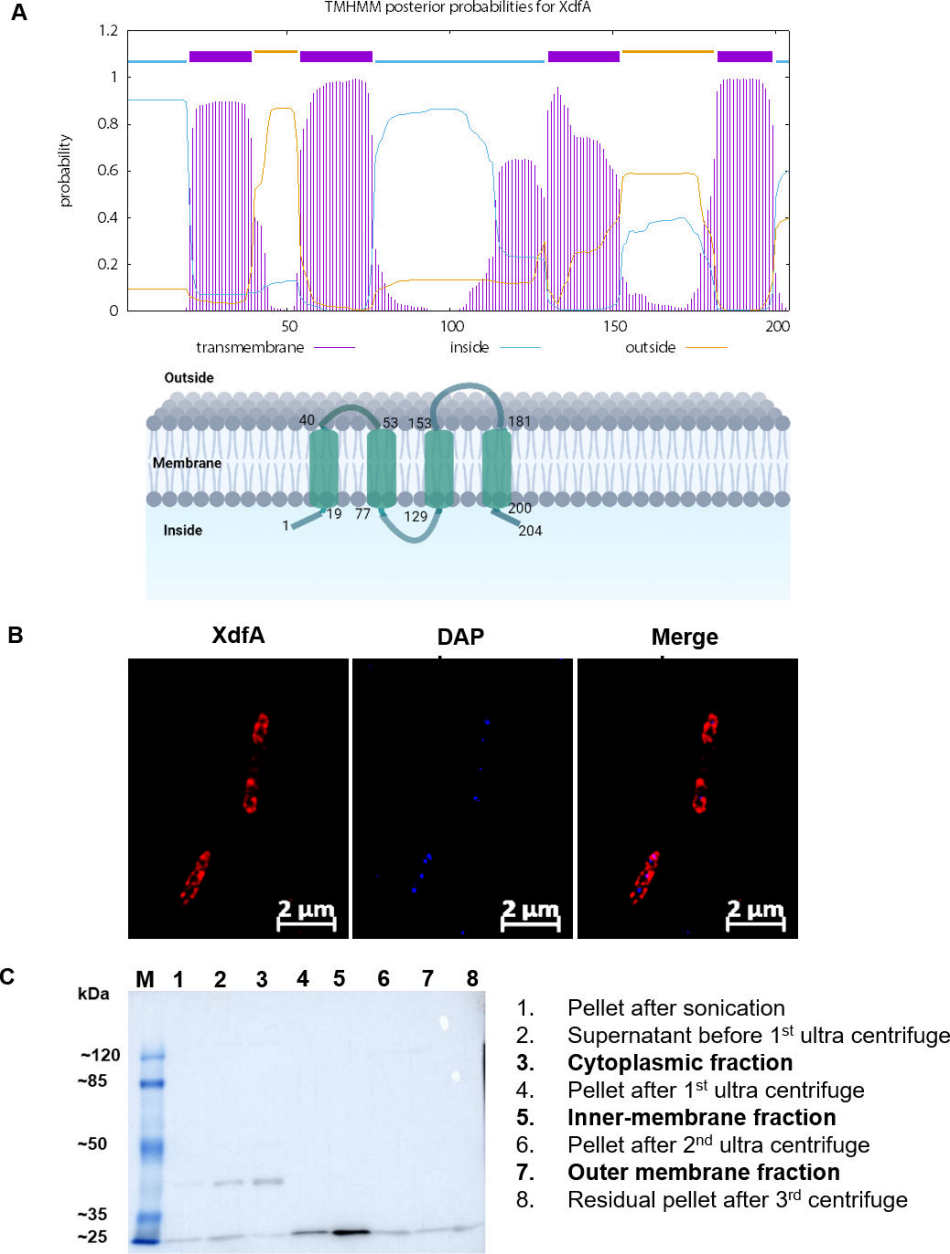
Transmembrane helix prediction and localization of XdfA. (A) Transmembrane helix prediction by TMHMM 2.0 (http://www.cbs.dtu.dk) suggested four membrane spanning helices, whereas both the N-terminal and C-terminal of the protein are protruding inside. The accompanying cartoon is showing the results obtained from the prediction, where the Arabic numerals are indicating amino acid residue number. (B) Representative anti-HA immunofluorescence images demonstrating localization of HA-tagged XdfA (red) into the membrane. DAPI (blue) was used to stain bacterial chromosome. (C) Western blot with anti-HA antibody after cell fractionation by ultracentrifugation. Wells 3, 5, and 7 contain cytoplasmic, inner membrane, and outer membrane fractions, respectively.

Based on preliminary amino acid homology analysis, it was predicted to be a putative SNARE-type Golgi protein of the DedA family. As a result, transmembrane helix prediction was carried out using the online tool TMHMM 2.0 (http://www.cbs.dtu.dk). This is a membrane protein topology prediction tool based on a hidden Markov model that effectively distinguishes between soluble and membrane proteins (25). Despite the fact that the protein’s N and C termini are both cytoplasmic, the results suggested four membrane spanning helices and two independent portions protruding outside (Fig. 5A). A hemagglutinin (HA) epitope tag was inserted at the C-terminus of XdfA to study the localization of this protein. Immunofluorescence microscopy was performed by interacting Alexa Fluor 594 conjugated secondary antibody against anti-HA antibody specific for XdfA-HA and observed under Structured Illumination Microscope (SIM). The SIM images are clearly showing the localization of XdfA into the peripheral membrane region of bacteria (red) distinct from the blue-stained 4',6-diamidino-2-phenylindole (DAPI)-bound chromosomal region (Fig. 5B). Bacterial membrane fractionation of the XdfA-HA tagged *Xcc* cells was performed to further validate our observation (26). To ensure that the membrane-fractionation is achieved, RNA polymerase Sigma-32, succinate dehydrogenase, and LPS were taken as markers for cytoplasmic, inner membrane, and outer membrane, respectively (data not shown). Western blot analysis using anti-HA antibodies detected the presence of a 30 kDa band in the inner membrane fraction of *Xcc* containing HA-tagged XdfA but not in either cytoplasmic or outer membrane fractions (Fig. 5C). This indicates that XdfA is an inner membrane protein.

### *Ab initio* structure prediction of XdfA

**Fig 6:**
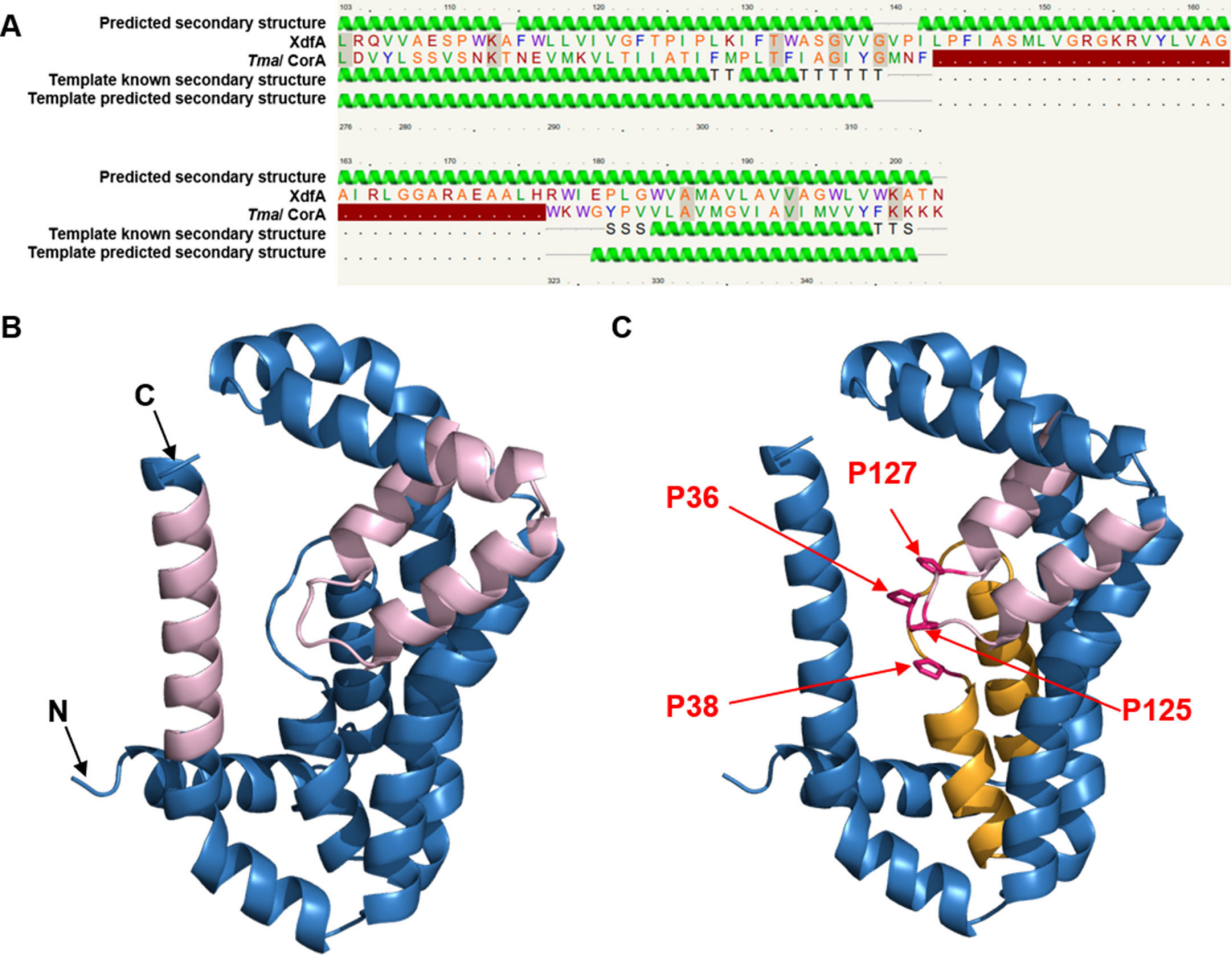
Bioinformatic structure prediction of XdfA. (A) Structural prediction using Phyre2 shows the highest 49% coverage (aa 103–203) alignment with CorA-like Mg^2+^ ion transporter of *Thermotoga maritima*. (B) The top-ranking *ab initio* predicted the structure of XdfA of *Xanthomonas campestris* pv. *campestris* using ColabFold. The total protein is colored sky blue, whereas regions homologous to *Tma*/CorA are colored light pink using PyMol. (C) Predicted model of XdfA showing two reentrant loops facing each other. Loop# 1 is colored bright orange, loop# 2 is colored light pink, and the proline residues where the loops have taken a turn are colored hot pink and marked by red arrows with respective residue numbers.

As it was difficult to solubilize XdfA for its purification to do other *in vitro* assays, we have taken the help of available *in silico* methods to deduce its predicted structure. Phyre2 is an online resource that uses high-throughput multiple sequence alignment to predict protein models and their putative functions from their primary amino acid sequence (27). The output report contained 10 such highly similar alignments. Out of them, highest 49% amino acid residue alignment of XdfA was found with the CorA-like Mg^2+^ transporter of *Thermotoga maritima* (Fig. 6A). To generate a high-quality 3D structure of XdfA, we have used ColabFold. It offers accelerated prediction of protein structure and complexes by integrating Mmseqs2’s fast homology search with AlphaFold2 or RoseTTAFold (28). Using PyMOL, the obtained protein was colored sky blue, while the homology region to TmaCorA was colored light pink (Fig. 6B). The *in silico* model also confirms the membrane topology of VMP1, TMEM41, and Tvp38 (VTT)/DedA domain, which contains two canonical transmembrane helices and two reentrant loops that face each other in the membrane (29). In addition to that, the reentrant loops contain helix-breaking proline residues between the two halves. Reentrant loop 1 turns near P36 and P38 whereas loop 2 turns near P125 and P127 (Fig. 6C). Transporters and ion channels with these reentrant loops include aquaporins, chloride channels, and solute carrier family proteins (30). Moreover, the reentrant loop 2 of XdfA is overlapping with the homologous CorA protein of *T. maritima*, which is a canonical ion transporter. Thus, the two anti-parallel reentrant loops may serve as a substrate-binding site for an ion-coupled receptor.

### XdfA regulates magnesium uptake and is necessary for growth under Mg-starvation

Bacterial magnesium transporters can be classified into three families, with CorA type transporters being the most prevalent. In the case of *Xcc* 8004, a homology search revealed the absence of MgtA type transporters but the presence of two paralogs of CorA, designated as *corA1* (XC_0628) and *corA2* (XC_1781). CorA1 shares 25% identity and 47% similarity in amino acid composition with its ortholog in *E. coli*, while CorA2 exhibits 28% identity and 44% similarity. Interestingly, these two paralogs display 31% identity and 51% similarity with each other. A comprehensive analysis of homology with other bacteria is provided in Tables S4 and S5.

**Fig 7:**
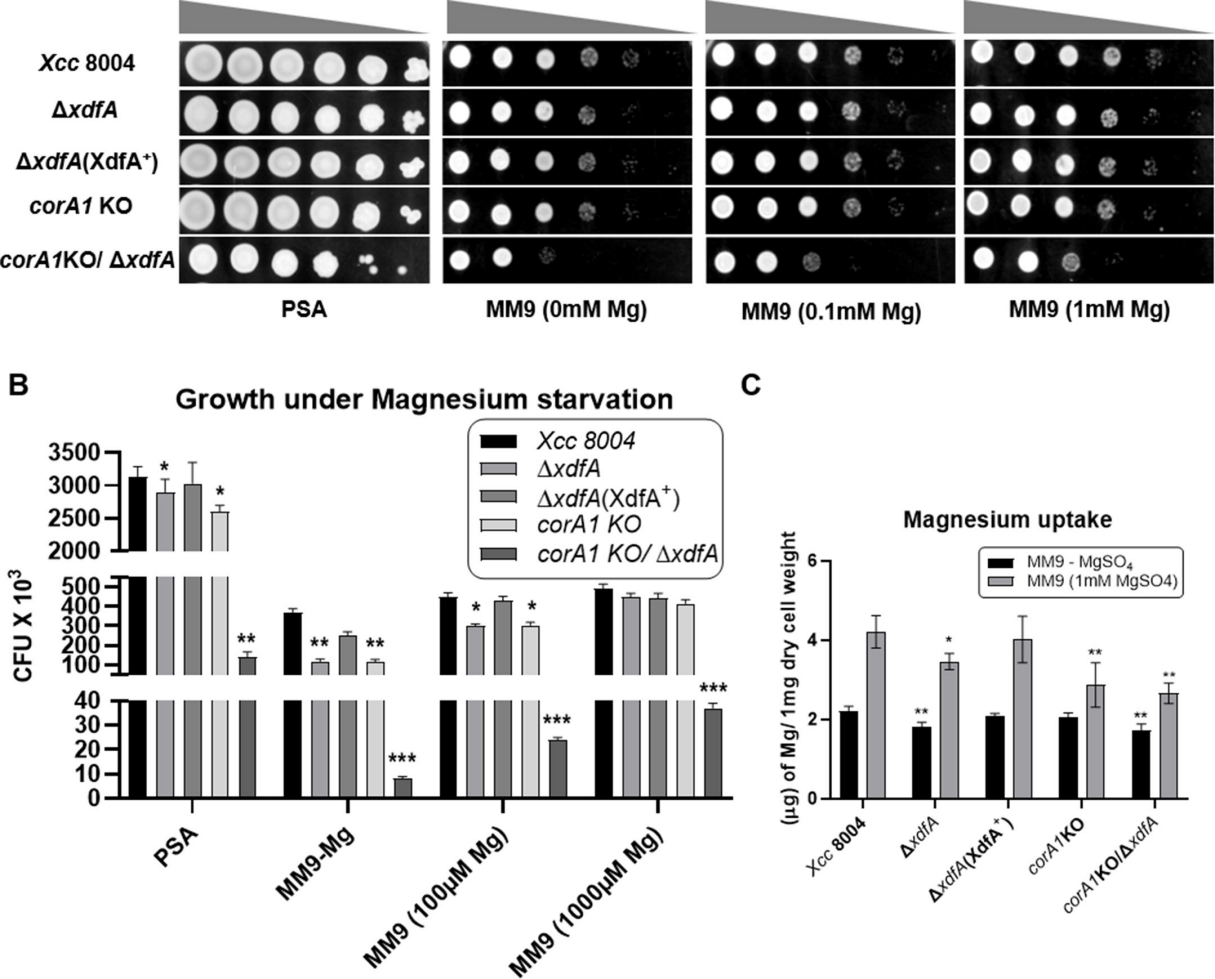
Growth and uptake of magnesium under differential magnesium conditions. (A) Dilution spotting images of different *Xcc* strains on PSA-rich media, MM9 minimal media without magnesium, and MM9 supplemented with 100 µM and 1,000 µM magnesium sulfate, 60 h post-inoculation. (B) Growth under PSA-rich media, MM9 without magnesium, supplemented with 100 µM and 1,000 µM magnesium sulfate. Thousand colony forming units (CFUs × 10^3^) were depicted as a single unit on the *Y*-axis. (C) Intracellular magnesium estimation of different *Xcc* strains in MM9 minimal media without magnesium and 4 h post-addition of 1 mM MgSO_4._ All these above experiments were performed in at least three biological replicates. Error bars represent the SD of the mean. *P* values were calculated using the Student’s *t* test. (*P < 0.05, **P < 0.01, and ***P < 0.001).

To gain a thorough understanding of magnesium homeostasis in *Xcc*, we conducted growth experiments under varying magnesium concentrations. Under nutrient-rich PSA media, all strains exhibited similar growth rates, except for the *corA1* knockout (KO)/∆*xdfA* double mutant, which displayed impaired growth. However, in magnesium-limited media without supplementation, the growth of the ∆*xdfA* and *corA1* KO strains was threefold lower compared to the wild-type *Xcc* 8004. In contrast, no discernible differences in growth phenotypes were observed between the ∆*corA2* and ∆*corA2*/∆*xdfA* mutants and *Xcc* under varying magnesium conditions (data not shown). Notably, the growth deficiency in the double mutant *corA1* KO/∆*xdfA* was 30-fold reduced. Supplementation of 100 µM MgSO_4_ partially compensated for this growth defect in the single mutants, albeit with a twofold difference compared to the wild type. However, the difference was amplified 10-fold for the double mutant. Further supplementation of 1 mM MgSO_4_ rescued the growth defect in the single mutants but failed to recover the deficiency in the *corA1* KO/∆*xdfA* mutant (Fig. 7A and B).

Further, we have estimated intracellular elemental magnesium content using atomic absorption spectrometry to assess any defect in magnesium uptake of *Xcc* 8004 in the absence of *xdfA*. Bacteria grown in rich media have no differences in intracellular magnesium content (Fig. S8A). However, after a 4-h-long magnesium quenching by growing the bacteria under MM9 without magnesium, when they were subjected to 1 mM MgSO_4_ supplementation, ∆*xdfA* was unable to uptake magnesium as effectively as wild-type *Xcc*. Both ∆*xdfA* and *corA1* KO were having 25% less intracellular Mg content, whereas *corA1* KO/∆*xdfA* was having 40% less (Fig. 7C). However, the elemental magnesium content of the cell-free supernatants collected during the experiment does not show any difference among them (Fig. S8B).

These findings suggest that, though XdfA is not the only protein responsible for magnesium transport and usage, it plays an important role in maintaining magnesium homeostasis of *Xcc* 8004.

## DISCUSSION

Proteins belonging to the DedA family are almost ubiquitous in reported microbial genomes (14) and there are more than 30,000 sequences available in the pfam database, which belongs to 8,000 species across kingdoms (Fig. S9). The members of the DedA family proteins have pleiotropic effects in the reported bacterial species *E. coli* and *Borrelia burgdorferi*. Mutants of DedA family proteins exhibit cell division defects (31, 32), sensitivity to alkaline pH, osmolytes (15) biocide sensitivity, and compromised proton-motive force (16, 17). But in our study, surprisingly, we have not found any difference in growth pattern of ∆*xdfA* under alkaline condition and in the presence of different osmolytes (Fig. S3 and S4). However, alternation in phospholipid moiety was observed (Fig. 4). Phospholipids constitute the inner leaflet, whereas LPS forms the outer leaflet of the outer membrane in Gram-negative bacteria (33). An endotoxin, lipid A, which is one of the constituents of LPS, plays significant role in bacterial pathogenicity and immune evasion (34). Though no significant alteration in the banding pattern of LPS was observed, the hypersensitivity response was too high in non-host benthi and tomato plants when these were infiltrated with ∆*xdfA* (Fig. S7). Transcriptional regulation of hypersensitive response-related genes was also found to be altered in case ∆*xdfA* (Fig. S7). Maintaining membrane homeostasis is of paramount importance for the growth of bacteria, both *in vivo* or *in vitro*. The compromised growth of ∆*xdfA in planta* might be a result of distorted membrane homeostasis (Fig. 2).

For an epiphytic pathogen like *Xcc*, adhesion to the host surface and successful entry are the primary steps of disease establishment. The ∆*xdfA* of *Xcc* was found to be compromised in surface attachment (Fig. 3) and *in planta* migration (Fig. 1; Fig. S2), which justifies its reduced virulence. Once inside host tissues, bacterial cells aggregate to raise the cell density to a critical mass to initiate and maintain interaction with host cabbage cells. Hence, the capacity to congregate and produce biofilms is a prerequisite for epiphytic survival and, thus, the potential for dispersal to a new niche, since it provides better protection against environmental stress, improved bacterial resistance against host defense responses, and antimicrobial tolerance (35). But, in our experiments, we found ∆*xdfA* to be deficient in biofilm formation in comparison to wild-type *Xcc* (Fig. 3), which is in congruence with our other results.

Magnesium is the most abundant divalent metal ion found in living system, having a bona fide functional and structural role. It acts as a cofactor in several metalloenzymes and also helps in maintaining the structures of membranes and ribosomes (36). In contrast to other metals, the intracellular level of magnesium remains high (0.5–2.0 mM), which need specific magnesium transporters (37). Three families of magnesium transporters named CorA, MgtE, and MgtA/MgtB have been identified in bacteria. In *Salmonella typhimurium*, CorA found to transport Mg^2+^ under normal Mg^2+^ concentration, whereas the energy expensive MgtA/MgtB-mediated transportation happens in an Mg^2+^-deprived condition (38). CorA and MgtE are ion-gated channels, while MgtA/MgtB is ATP hydrolysis dependent. The limiting extracellular magnesium level activates PhoQ/*P* two-component system to expedite MgtB-mediated Mg^2+^ transport (39, 40). The Mg^2+^-induced PhoP/PhoQ system is required for virulence in a variety of bacterial pathogens, starting from enteric pathogens *Salmonella* and *Shigella* to plant pathogen *Erwinia carotovora* (41). Nearly all reported bacteria were found to have multiple Mg^2+^ transporters; however, the role of alternative magnesium transporters beyond the abovementioned three families of proteins remains obscure.

The DedA family of proteins have been proposed to harbor membrane- spanning domains similar to membrane-bound transporters having essential charged amino acids embedded in the membrane, which helps in the maintenance of proton motive force in case of *E. coli* (42). Our multiple sequence alignment results also provide evidence about the evolutionary conservation of glutamic acid residues across plant pathogens (Fig. 1). Additionally, *in silico* topology prediction followed by immune fluorescence microscopy and western blot analysis establish that *Xcc*/XdfA is an inner membrane bound protein (Fig. 5). To our knowledge, till now there have been only two reports, where DedA family proteins are reported to be involved in heavy metal transport. In *Ralstonia metallidurans* (renamed as *Cupriavidus metallidurans*), it was linked to selenite uptake (43), whereas, in the case of *Rhodanobacter* sp., it was found to be responsible for expulsion of indium (44). However, high-throughput multiple sequence alignment experiments using *Xcc*/XdfA residues showed homology toward CorA-type magnesium transporter of *Thermotoga maritima*. High-resolution structural prediction using ColabFold confirmed the presence of two characteristic anti-parallel hairpin-like non-transmembrane region along with two *trans*membrane helices. These kinds of oppositely faced reentrant loops are the signature property of many transporters, often credited for ion-translocation function (30, 45).

The growth deficiency exhibited by ∆*xdfA* is nearly equal to the canonical magnesium transporter mutant *corA1* KO. But when both these genes were mutated, the growth deficiency was significantly higher compared to the single mutants (Fig. 7A and B). The functional redundancy of the single mutants signifies the essentiality of magnesium for the growth and survival of bacteria. Intracellular magnesium uptake of ∆*xdfA* and *corA1* KO was significantly less in comparison to wild-type *Xcc*, whereas the uptake deficiency due to double mutation was even higher (Fig. 7C). But in none of the cases, the uptake was completely abolished indicating the role of other players in maintaining magnesium homeostasis apart from *xdfA* and *corA1*, which are yet to be identified, . Having alternate transporters for specific macromolecules emphasizes the essentiality of the metal atom for the survival of bacteria. Maintaining optimum Mg^2+^ concentration is crucial as it acts as the central metal ion of various enzymes as well as stabilizing ribosomes and neutralizing nucleic acids (41). It also neutralizes phospholipid head groups and surface molecules outside the cytoplasm. The altered phospholipid pattern observed in the thin layer chromatography experiment (Fig. 4) might be an explanation for skewed Mg^2+^ homeostasis in case of ∆*xdfA*.

**Fig 8:**
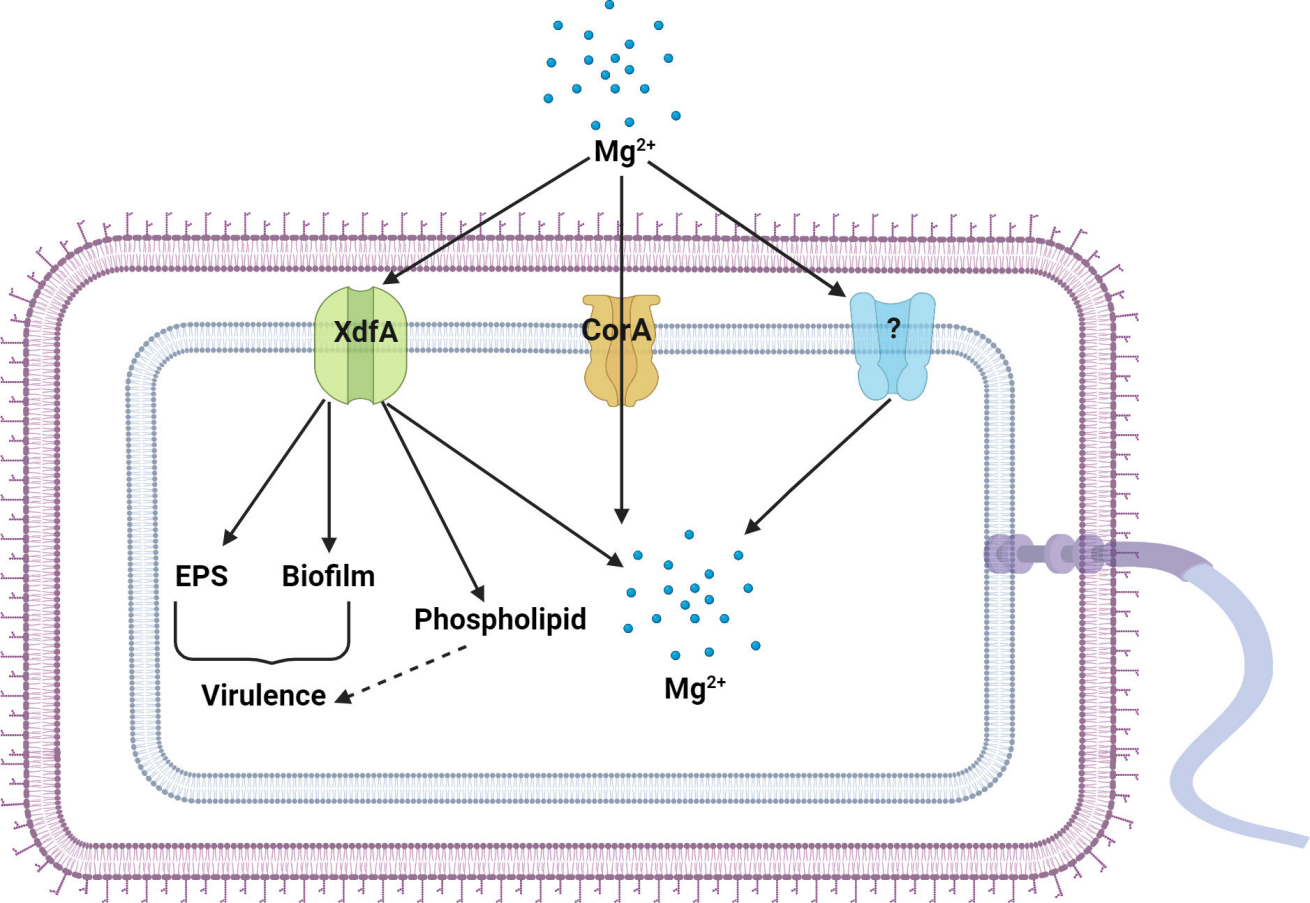
A proposed model for the role of XdfA in the regulation of magnesium homeostasis and other virulence factors. XdfA is an inner membrane channel protein that has a role in magnesium uptake along with the canonical magnesium transporter CorA and a few unknown transporters. XdfA also positively regulates other virulence and membrane associated traits like biofilm formation, EPS production, and phospholipid production, which ultimately result in virulence deficiency.

Based on the above observation, we are proposing a working model for the role of XdfA in magnesium homeostasis and other virulence factors (Fig. 8). We discovered that XdfA plays a role in pathogenicity-related features such as EPS production and biofilm formation, which are connected to the results of virulence assay carried out on the host cabbage plant. As this is an inner membrane protein that was established from our experimentation, we checked its membrane homeostasis functions as well as phospholipid profiling, where we found that ∆*xdfA* is producing phospholipids with altered compositions. This might compromise its chances of surviving inside the host plant. We are also concluding that XdfA has a role in magnesium uptake due to which, in the absence of it, growth is stunted in magnesium starvation condition.

This study thus explains the prominent role of XdfA in the maintenance of membrane homeostasis by optimizing the secretion of EPS and phospholipids in *Xcc*. Additionally, the study throws light on the essentiality of the gene *xdfA* for optimum virulence in *Xanthomonas campestris* pv. *campestris*. It also provides first experimental evidence of a DedA family protein to be acting as an alternate magnesium uptake protein in case of a plant pathogen.

## MATERIALS AND METHODS

### Bacterial strains, plasmids, and growth conditions

*Xanthomonas campestris* pv. *campestris* (*Xcc* 8004) was used as a parental strain. *Xcc* and its derived strains were maintained on PSA plates, or grown in peptone sucrose (PS) broth supplemented with appropriate antibiotics (46) at 28°C with shaking at 200 rpm (New Brunswick Scientific, Innova 43, Edison, NJ, USA). *Escherichia coli* strains were grown at 37°C in Luria-Bertani (LB) medium with shaking at 200 rpm or on LB agar plates with appropriate antibiotics (47). Detailed information on the bacterial strains and plasmids used in this study is listed in Table S2. The concentration of antibiotics used was as follows: rifampicin (Rif; 50 µg/mL), spectinomycin (Spec; 50 µg/mL), kanamycin (Kan; 50 µg/mL), ampicillin (Amp; 100 µg/mL), gentamycin (Gent; 5 µg/mL), nalidixic acid (Nal; 50 μg/mL), and 5-bromo-4-chloro-3-indolyl-d-galactoside (Xgal; 25 μg/mL).

### Molecular biology techniques

Standard molecular biology techniques were performed for genomic DNA isolation, plasmid isolation, restriction digestion, ligation, transformation, and agarose gel electrophoresis, as described previously (48). Plasmid DNA was isolated using the Qiagen Plasmid Midi Kit (Qiagen, Cat No./ID: 12143) according to the manufacturer’s instructions. Thermo Scientific NanoDrop 2000 spectrophotometer (Thermo Fisher Scientific, Waltham, MA, USA) was used to check the concentration and purity of the DNA. PCR was performed using High-fidelity Phusion Taq polymerase (Thermo Fisher Scientific, Waltham, MA, USA); furthermore, restriction digestions and ligations were performed with the respective enzymes (New England Biolabs, Ipswich, MA, USA) according to the manufacturer’s instructions. QIAquick Gel extraction kit (Qiagen 28704) was used for the extraction of DNA from agarose gels. DNA transformations were performed either by heat shock or electroporation. The oligonucleotide primers used in this study are listed in Table S3. SDS-PAGE, polyacrylamide gel staining, and western blot experiments were carried out as described previously (49).

### Bacterial mutant and complemented strain generation

The *xdfA* (XC_2523) and *corA2* (XC_1781) genes in *Xcc* 8004 were deleted utilizing pK18mobSacB system, a suicidal vector, harboring the kanamycin resistance gene (kan^r^) and the SacB gene as selection and counter-selection markers, respectively, as described earlier (50). The oligonucleotide primers used for constructing the deletion strains are listed in Table S3. Complementing strain [Δ*xdfA*(Xdf^+^)] was generated by reconstituting the full-length gene *xdfA* into the genomic background of the full-length deletion mutant (Δ*xdfA*) by using the pK18mobSacB system as described earlier (51). The insertional nonpolar mutant of *corA1* (XC_0628) was obtained by amplifying 348 bp internal fragment of *corA1* by primers listed in Table S2, and then cloning it to the suicidal vector pK18mob to get *corA1* KO having kan^r^, as described earlier (8). A double mutant *corA1* KO/Δ*xdfA* was obtained by electroporating the same construct into the Δ*xdfA* background.

### Phylogenetic dendrogram

After amino acid sequence alignment with ClustalOmega, a phylogenetic dendrogram of XdfA homologs in the NCBI database was constructed using the PRESTO method (https://ngphylogeny.fr/) (52). *Escherichia coli* (Eco; P0ABP6), *Pseudomonas aeruginosa* (Pae; WP_144125346.1), *Xylella fastidiosa* (Xfa; WP_004088189.1), *Xanthomonas campestris* pv. *campestris 8004* (*Xcc*; WP_011036885.1), *X. vesicatoria* (Xve; WP_005992402.1), *X. arboricola* (Xar; WP_115043467), *Xanthomonas axonopodis* (Xax; WP_078586924.1), *X. citri* pv. *citri* (Xac; CEG16020.1), *Xanthomonas oryzae* pv. *oryzae* (Xoo; WP_011259521.1), and *X. oryzae* pv. *oryzicola* (Xoc; WP_014503663.1) from the NCBI database were used as input.

### Virulence, *in planta* survivability, and migration assay

The clip inoculation method was used to infect 45-day-old Indian Super Hybrid cabbage plants. For clip inoculation, *Xcc* strains were grown till saturation and normalized to a cell number of approximately 10^9^ cells/mL. The tip of approximately 25 leaves per strain were gently incised, and the average lesion length was measured at 15 dpi. The *in planta* survivability assay was performed at 3 dpi, 7 dpi, and 15 dpi to represent the early, middle, and late stages of infection, respectively. After surface sterilization with sodium hypochlorite and 70% ethanol, 1 cm^2^ of cabbage leaves was crushed with a sterile mortar pestle. This was serial diluted and plated on PSA plates to record CFUs. For the migration assay, cabbage leaves were surface sterilized in a similar fashion and approximately 1 cm thick slices were cut to place them over PSA plates to record any growth. All these experiments were repeated thrice, and the mean values were plotted with error bars representing standard deviations.

### Isolation and quantification of EPS

Extracellular polysaccharide (EPS) was isolated from the *Xcc* strains by acetone precipitation method, as described previously (53). In brief, 20 µL of overnight grown culture were spotted on PSA plates and incubated at 28°C for 3 days. These spots were scraped, resuspended in PBS, and then centrifuged at 8,000 *g* for 10 min to get cell-free supernatant. Before pelleting the cells, 100 mL was aliquoted for serial dilution plating to determine CFUs. The EPS present in the cell-free supernatant was precipitated by adding formamide and two volumes of ice-cold acetone, and then incubated at 4°C overnight. The following day, the tubes were centrifuged at 7,969 *g* for 20 min, and the pellet was dried before measuring the total carbohydrate content with a phenol-sulfuric acid colorimetric method using D-glucose as the standard. The tests were carried out in triplicate.

### Phospholipid analysis

The *Xcc* 8004, ∆*xdfA*, and ∆*xdfA* (XdfA^+^) strains, as well as the *E. coli* (WT) strain, were grown to late log phase and normalized to an OD_600_ of 0.8. The phospholipids were extracted by first subjecting the OD-normalized bacterial cultures to 12.5 mM of pH 4.4 sodium acetate at 100°C for 30 min, followed by lipid extraction in the Bligh Dyer mixture, as described previously (54). The lipid samples were lyophilized and then dissolved in 100 µL of a 2:1 chloroform-methanol mixture at the end of each extraction protocol. Ten microliters of these samples along with the *E. coli* total lipid standard (Sigma-Aldrich, St. Louis, MO, USA) were then spotted on the TLC plates. The phospholipid extracts were developed in chloroform, methanol, water, and ammonia solvent (65:25:3:6:0.4, vol/vol/vol/vol). Phospholipids on the TLC plate were then visualized by spraying with 10% sulfuric acid in ethanol followed by charring at 200°C.

### Static biofilm and attachment assay

Biofilm assay was carried out exactly as described previously (8). In brief, different *Xcc* strains were grown overnight in PS broth medium supplemented with the required antibiotics at 28°C and 200 rpm, centrifuged, washed, and resuspended in sterile PBS. Approximately 1 × 10^9^ cells were transferred into 4 mL of fresh PS medium in 12-well sterile polystyrene culture plates and incubated at 28°C without shaking. After 48 h, the medium was gently decanted, and the wells were washed with autoclaved Milli-Q water, followed by staining with 0.1% crystal violet. Excess stain was removed by washing, and the absorbance of the attached cells dissolved in 90% ethanol was measured at 570 nm.

For the attachment assay, cells were grown in 12-well polystyrene plates as biofilm assay. After washing the wells, attached cells were resuspended in PBS (pH 7.4) and dilution plated on PSA plates to quantify cell attachment.

### Protein topology prediction

Amino acid sequence of XdfA was collected from the Integrated Microbial Genomes and Microbiomes site (IMG/M: https://img.jgi.doe.gov/m/) (55). This sequence was submitted into the query box provided in the online membrane protein topology prediction tool TMHMM-2.0 (http://www.cbs.dtu.dk/services/TMHMM/) (25). This prediction method is based on a hidden Markov model, which can effectively discriminate between soluble and membrane proteins. The result provided information about the total number of transmembrane helices and their in/out orientation relative to the membrane along with the specific residue number, based on which a cartoon was drawn.

### Immunofluorescence microscopy

Immunofluorescence staining was performed as described previously (56) with few modifications. Briefly, 9 mL methanol was added to 1 mL OD_600_ 0.4 bacterial culture and incubated on ice for 40 min. Eight hundred microliters of 4% formaldehyde was added to this and incubated for 10 min, followed by a spin at 4,000 *g* for 5 min. One microliter of methanol was added to the pellet from this spin to resuspend, and then 20 µL of the fixed cells was spread evenly on a poly-l-lysine (Sigma-Aldrich P8920) coated coverslip. Twenty microliters of lysozyme (Sigma-Aldrich 62971; 2 mg/mL) was put on the coverslip for permeabilization, followed by two times wash with PBS. Cells were incubated in a blocking buffer containing 2% BSA, before washing twice with phosphate buffered saline with 0.5% Tween 20 (PBST). For primary antibody staining, cells were incubated with 100 µL (1:400) rabbit anti-HA primary antibody (Abcam ab9110) for 1 h, followed by three washes with PBST with a 5-min incubation for each wash. In the secondary antibody staining, cells were incubated with 100 µL (1:1,000) goat anti-rabbit IgG secondary antibody, Alexa Fluor 594 (Thermo Fisher Scientific A32740) for 40 min, followed by three washes with PBST with a 5-min incubation for each wash. Then the coverslip was treated with 100 µL DAPI (1:200) for 2 min, followed by three washes with PBST. Twenty microliters of 80% glycerol was put on the slide before putting on the coverslip and sealing it with nail polish. Imaging was performed on a super-resolution microscope (Zeiss SIM with 63× oil immersion objective) at excitation and emission wavelength settings of 590 and 618 nm, respectively. Raw images were processed with the aid of ZEN Blue 3.3 software (Zeiss microscopy portal).

### Sub-cellular fractionation

XdfA localization study was performed as described earlier (26). Bacterial cultures grown in peptone sucrose broth were pelleted and resuspended in buffer containing 5 mM Tris-Cl (pH 8.0), 0.375 M sucrose, 1 mM EDTA, and 30 mg/mL lysozyme. The cell pellet was sonicated and centrifuged at 5,000 rpm for 10 min. The supernatant was centrifuged repetitively at 6,000 rpm for 8 min until unlysed cells were removed. The supernatant was centrifuged at 90,000 rpm for 2 h in ultracentrifuge. The supernatant after the first ultracentrifuge was retained as a cytoplasmic fraction. The pellets were resuspended in Triton X-100 buffer containing 10 mM Tris-Cl (pH 8.0), 1% Triton X-100, and 5 mM MgCl_2_, and incubated for 30 min at RT, followed by ultracentrifugation at 70,000 rpm for 30 min. The supernatant after the second ultracentrifuge was retained as the inner membrane fraction, and the pellet was resuspended in buffer containing 50 mM Tris-Cl (pH 8.0), 10 mM EDTA, and 1% Triton X-100 and incubated at RT for 30 min, followed by centrifugation at 10,000 rpm for 1 h. The supernatant was collected as outer membrane fraction. Protein-normalized samples from all the fractions were loaded on 12% SDS-PAGE and stained with Coomassie brilliant blue to recheck the protein normalization, and accordingly, the volumes were corroborated based on the observation of the band intensities. All the eight fractions, collected in various steps of the process, were solubilized in 1× SDS-PAGE sample buffer, heated for 5 min at 98°C, and separated on 12% acrylamide gels. The samples were transferred to polyvinylidene difluoride membranes by the wet-transfer method, and Western blot analysis (49) was done using rabbit anti-HA primary antibody (Abcam ab9110). The secondary antibody was goat anti-rabbit IgG conjugated to alkaline phosphatase (Thermo Fisher 31460). Detection was performed using chromogenic substrates as described by Sambrook et al. (48).

### Molecular modeling of XdfA

Predicted models of DedA were obtained using online resources such as Phyre2 (27). For the Phyre2 prediction, the amino acid sequence of protein DedA was submitted as the query sequence box provided in the online resource (http://www.sbg.bio.ic.ac.uk/phyre2). Intensive modeling mode was selected, and the sequence was submitted for prediction. The top-scoring model in Protein Data Bank (PDB) format was available upon completion of the job. A detailed report having alignment and percentage coverage data had also been obtained from this process.

ColabFold from Google Colaboratory (https://colabfold.mmseqs.com/) was used to predict a three-dimensional model of the protein XdfA. This open resource platform provides expedited prediction by combining fast homology search by MMSeqs2 with AlphaFold2 or RoseTTAFold (28). The .pdb file generated from this prediction was visualized and analyzed for color denotation using PyMOL (57).

### Intracellular magnesium estimation

The intracellular magnesium content was measured from different *Xcc* strains as described previously for iron (12) with few modifications. Briefly, overnight grown saturated culture was normalized to OD_600_ 0.2 and grown further in modified minimal media MM9 without magnesium for another 4 h to induce starvation, followed by a supplementation of 1 mM MgSO_4_. Four-hour post-supplementation, the cells were harvested by centrifugation, washed twice with MM9 without magnesium, lyophilized, and their dry weight measured. Further, the lyophilized cells were dissolved in 30% HNO_3_ at 80°C overnight and then diluted 50-fold with sterile double-distilled water. The cellular magnesium was determined by using atomic absorption spectrometer (iCE 3300 AAS by Thermo Fisher Scientific) against the elemental Mg-standards provided by the manufacturer.

### Growth under magnesium-deprived condition

To check growth under magnesium starvation condition, the wild-type *Xcc* along with Δ*xdfA*, Δ*xdfA* (XdfA^+^) along with *corA1* KO and *corA1* KO/Δ*xdfA* were grown overnight in rich PS broth until the late logarithmic phase. Then the cultures were washed twice with MM9 without magnesium, normalized to OD_600_ 1.0, followed by serial dilution up to 10^5^-fold with that media. Serially diluted cells (2.5 μL) were then spotted on to PSA along with MM9 devoid of Mg and MM9 supplemented with 100 µM and 1,000 µM MgSO_4_ agar plates. The plates were incubated at 28°C for 60 h before taking observation.

### Statistical analysis

All the wet lab experiments except bio-informatic analysis have been performed at least thrice with three biological replicates. Graphs, calculations, and statistical analyses were performed using GraphPad Prism software version 8.0 for Windows (GraphPad Software, San Diego, CA, USA). A paired two-tailed Student’s t test was used for pairwise comparisons. *P* values of <0.05 were considered statistically significant.

## Data Availability

The data that support the findings of this study are available in the supplemental material of this article and also are available from the corresponding author upon reasonable request.

## SUPPLEMENTAL MATERIAL

Supplemental Figures mbio.01361-23-s0001.pdf Fig. S1 to S9.

Supporting Text and Tables mbio.01361-23-s0002.docx Tables S1 to S6, supporting methods, and supplemental figure legends.

ASM does not own the copyrights to Supplemental Material that may be linked to, or accessed through, an article. The authors have granted ASM a non-exclusive, world-wide license to publish the Supplemental Material files. Please contact the corresponding author directly for reuse.
