# Supplementary material for: XdfA, a novel membrane-associated DedA family protein of Xanthomonas campestris, is required for optimum virulence, maintenance of magnesium, and membrane homeostasis
Source: mBio. 2023 Jul 27;14(4):e01361-23. doi: 10.1128/mbio.01361-23 (PMC10470534; doi:10.1128/mbio.01361-23)
Supplement: Supplemental Figures — Fig. S1 to S9. [file mbio.01361-23-s0001.pdf]

Fig S1

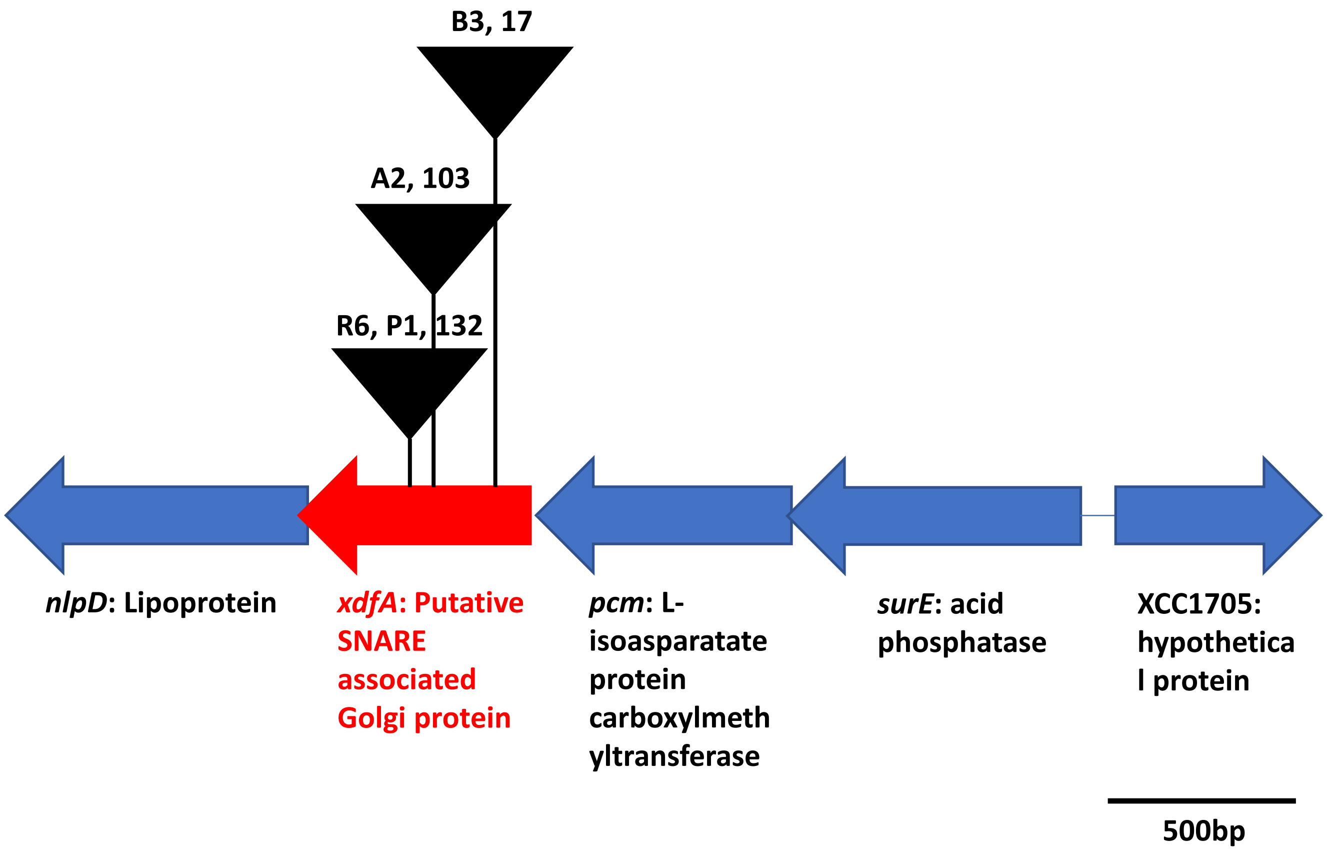

Figure S1. Schematic representation of *xdfA* with its neighbourhoods. Three independent Tn5 transposon insertion sites (represented by inverted triangles) are found at the corresponding amino acid residue 17, 103, and 132 in the *xdfA* locus (red coloured arrow). A length of 1 cm corresponds to 200 nucleotide bases in the schematic representation of the genomic organization in *Xcc* 8004 for the genes *xdfA* and its neighboring genes (blue colored arrow).

Fig S2

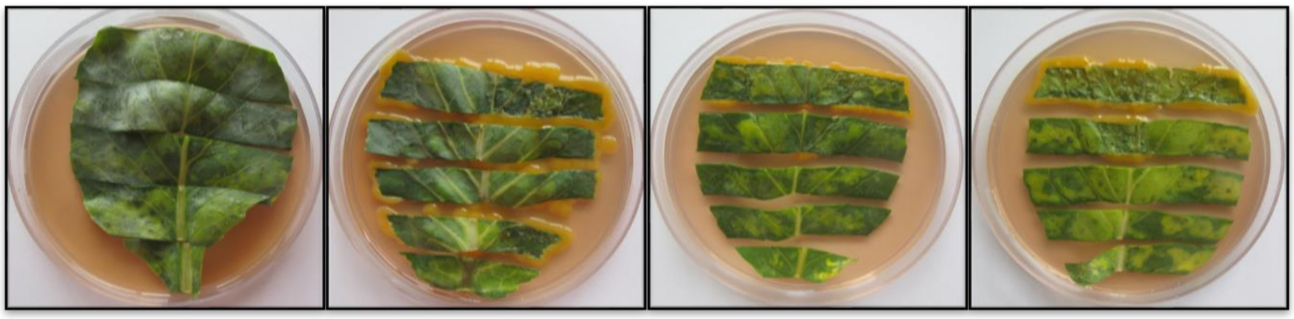

Figure S2: Bacterial migration inside host cabbage plant. Representative image of PSA plates showing *in planta* migration of *Xcc* strains, three days post inoculation.

Fig S3

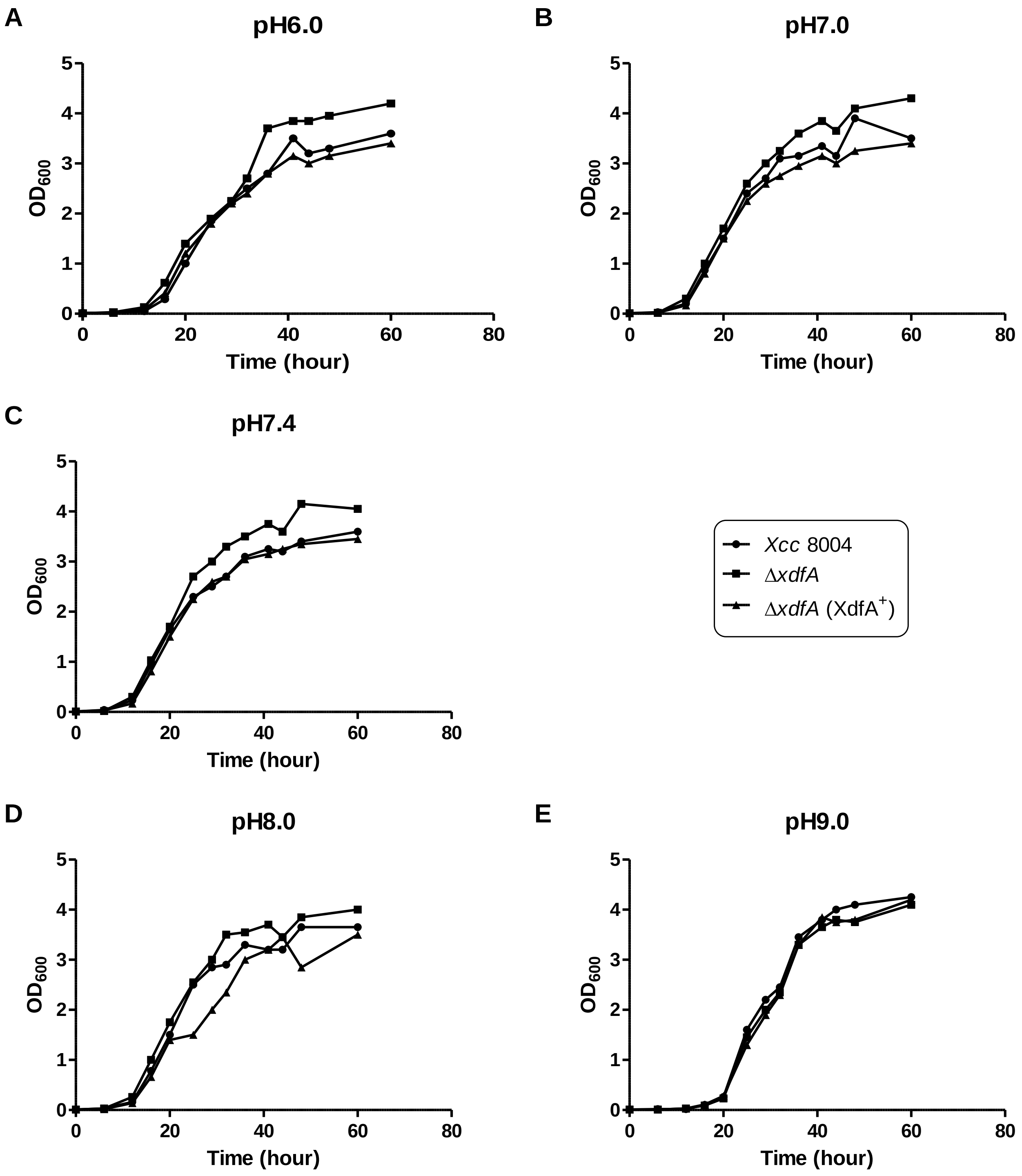

Figure S3: Growth phenotype under altered pH conditions. Wild type *Xcc* 8004, Δ*xdfA* and Δ*xdfA*(XdfA^{+}) were grown in rich PS broth in (A) pH 6.0, (B) pH 7.0, (C) pH 7.4, (D) pH 8.0, and (E) pH 9.0. The cultures were grown at 28°C and growth phenotypes are recorded as absorbance till 60th hour post inoculation.

Fig S4

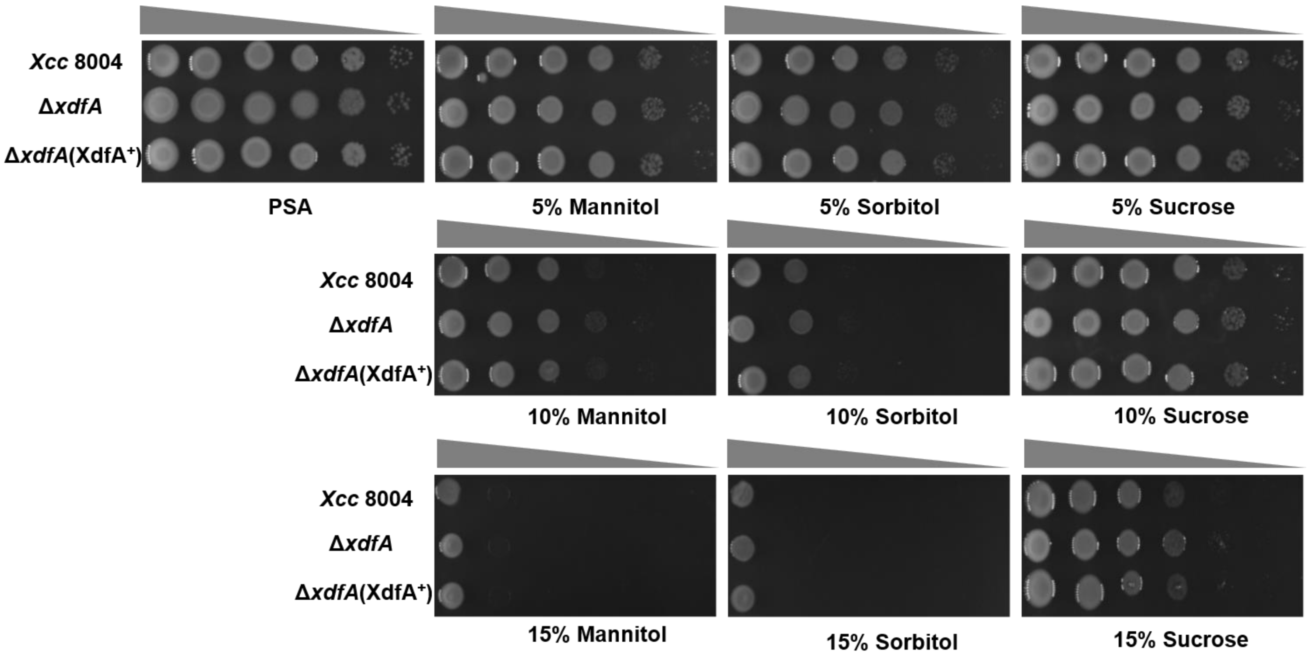

Figure S4. Growth phenotype in presence of osmolytes. The strains- *Xcc* 8004, Δ*xdfA* and Δ*xdfA*(XdfA$^+$) were grown till mid log phase and normalized to OD_{600} 1.0, followed by serially dilution and spotting on PSA plates supplemented with 5%, 10% and 15% of Mannitol, Sorbitol and Sucrose. The experiment was performed in three biological replicates.

Fig S5

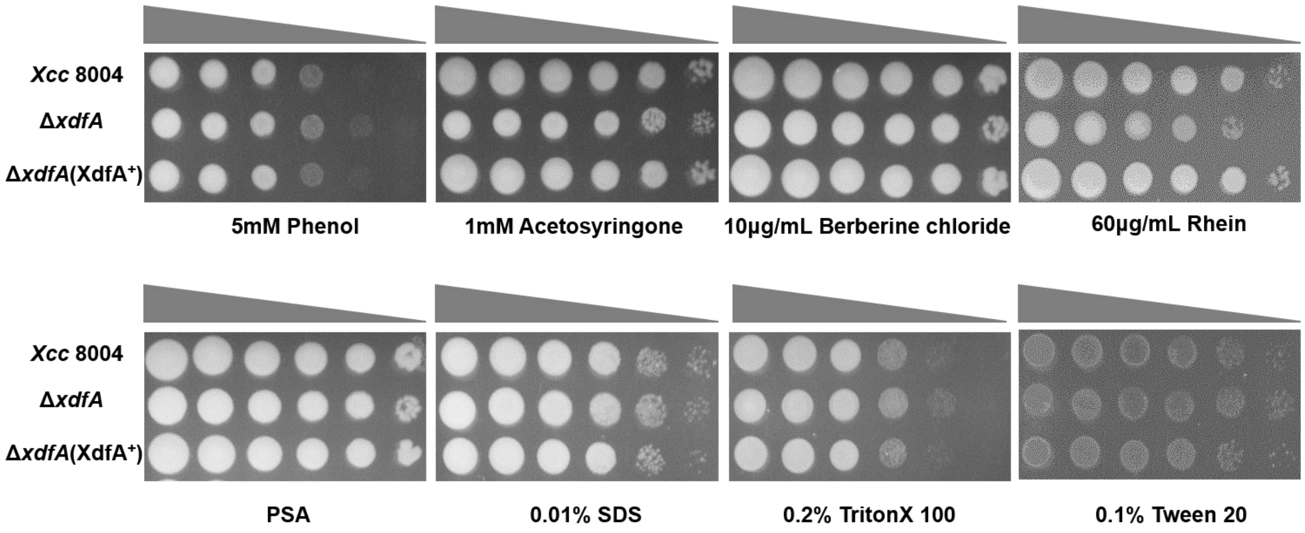

Figure S5. Growth in presence of Phenolics and detergents. Different Xcc strains were grown till mid log phase and normalised to OD_{600} 1.0 before serially diluted and spotted on PSA plates supplemented with 5mM Phenol, 1mM Acetosyringone, 10µg/mL Berberine chloride, 60µg/mL Rhein (top panel) 0.01% SDS, 0.2% Triton X 100 and 0.1% Tween 20 (bottom panel). The experiment was performed in three biological replicates.

Fig S6

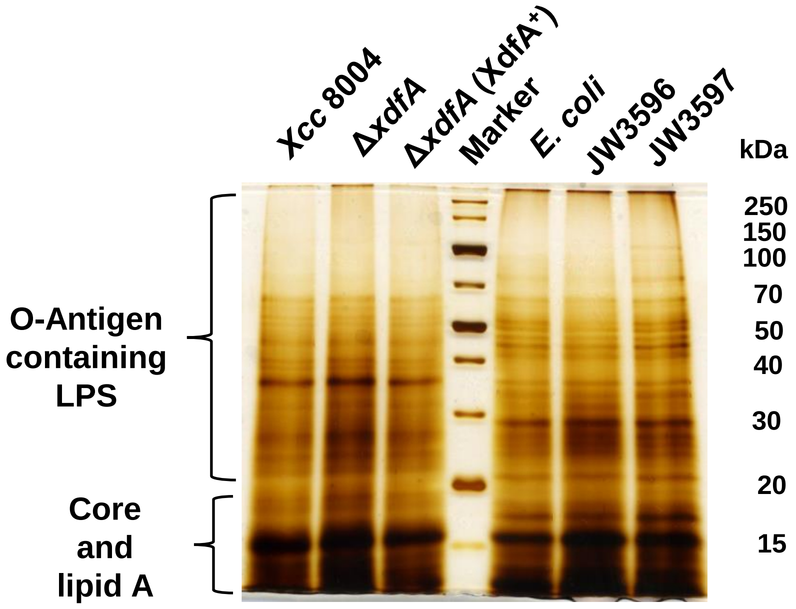

Figure S6. Silver-stained Lipopolysaccharide profiling of the LPS mutant JW3596, JW3597, wild type *E. coli* along with *Xcc* 8004, Δ*xdfA* and Δ*xdfA*(XdfA^{+}) on 12% SDS-PAGE. Boxes are drawn to indicate the differential banding pattern. These experiments were performed as three biological replicates.

Fig S7

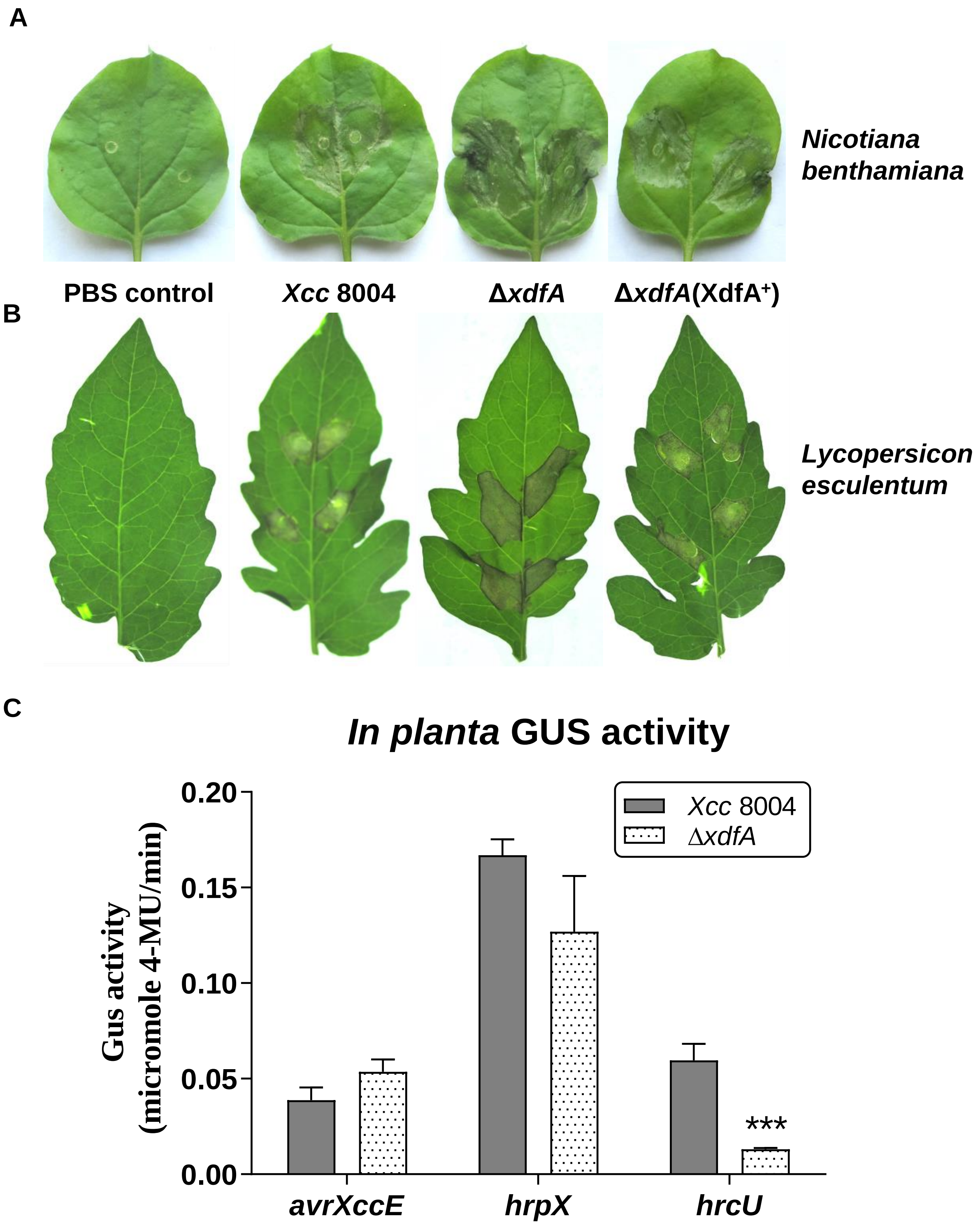

Figure S7. Hyper sensitivity response in nonhosts and transcriptional regulation of HR genes. (A-B) *Nicotiana benthamiana* and *Lycopersicon esculentum* plants showing hypersensitive response 3 days post infiltration with different *Xcc* strains. (C) *in planta* GUS reporter assay with different *hrp* gene promoter expression pattern inside cabbage host. Error bars represent SD of the mean. *P* values were calculated using Student's *t* test. (***p < 0.001).

Fig S8

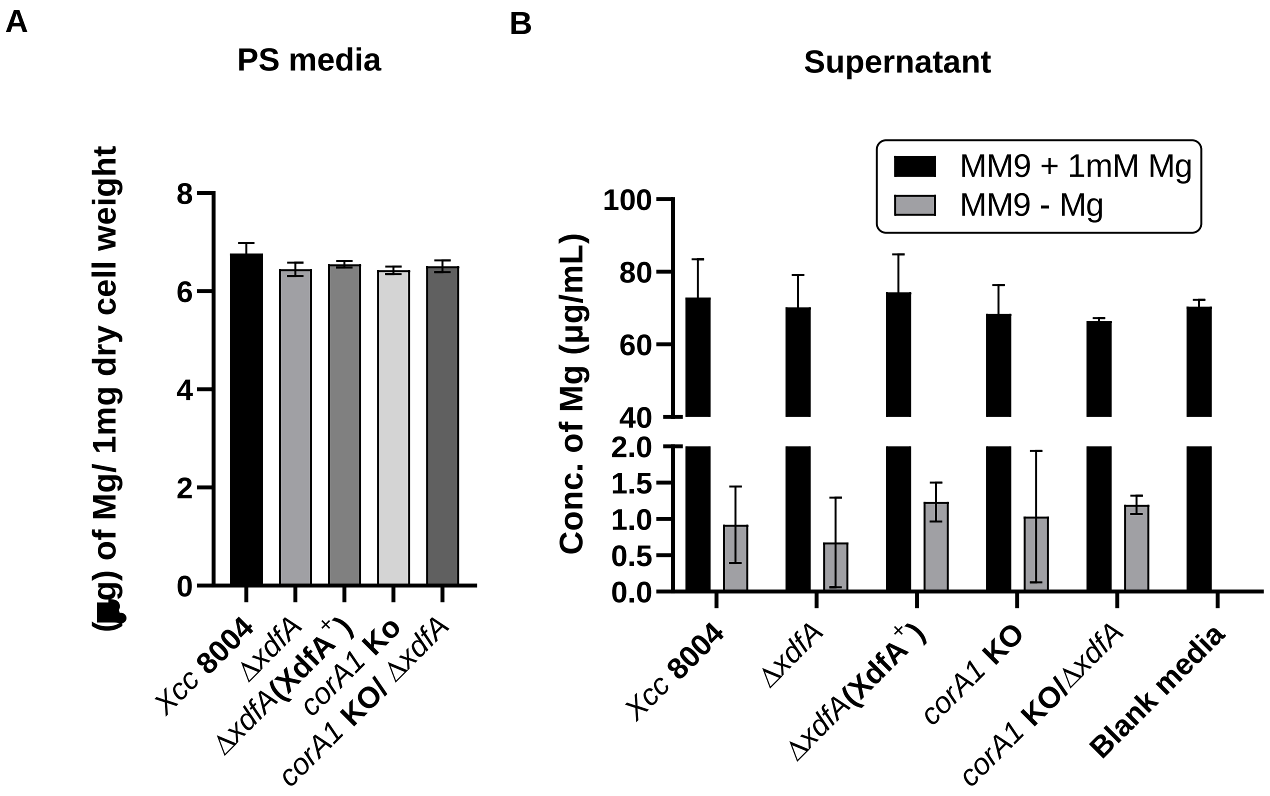

Figure S8. Elemental magnesium content measured by atomic absorption spectrometry (AAS). (A) Intracellular magnesium content measured from the lyophilized cell pellet by AAS after growing the cells in rich PSA medium (B) Elemental magnesium content of the cell free supernatant and the media, measured by AAS after growing the cells for eight hours with or without supplementing 1mM $MgSO_4$ to MM9 minimal media. All the experiments have been performed with three biological replicates.

Fig S9

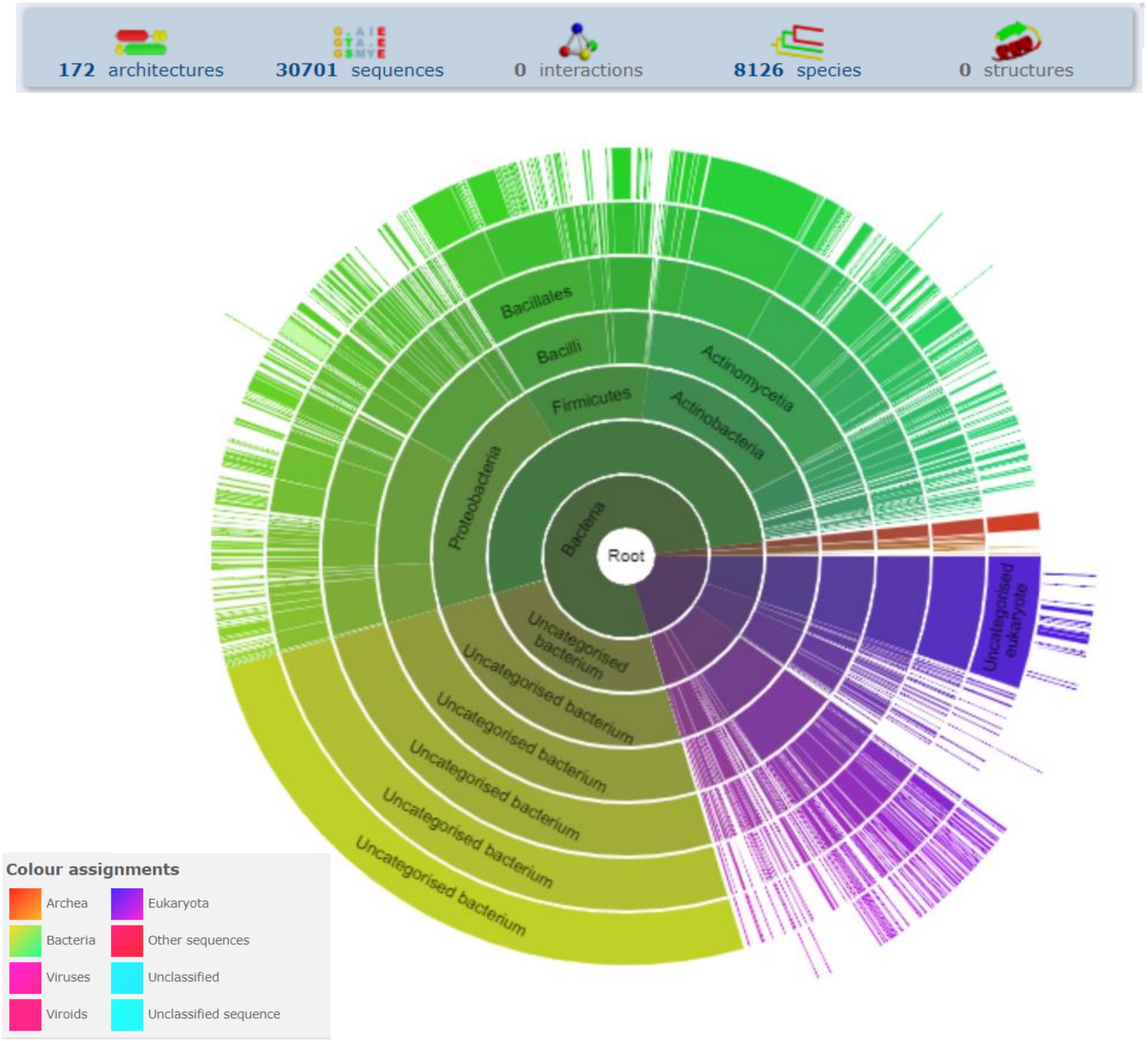

Figure S9. DedA is a ubiquitous protein group with no known structures or interactions. Modified "sunburst" representation of the species tree for the DedA family of proteins (*SNARE_assoc* [PF09335]) generated using "pfam" domain of xfam.org. It is a simple graphical representation of the family across species. Each node in the tree is represented as a separate arc, arranged radially with the super kingdoms in the centre and the species arranged around the outermost ring.
