# Supplementary material for: XdfA, a novel membrane-associated DedA family protein of Xanthomonas campestris, is required for optimum virulence, maintenance of magnesium, and membrane homeostasis
Source: mBio. 2023 Jul 27;14(4):e01361-23. doi: 10.1128/mbio.01361-23 (PMC10470534; doi:10.1128/mbio.01361-23)
Supplement: Supporting Text and Tables — Tables S1 to S6, supporting methods, and supplemental figure legends. [file mbio.01361-23-s0002.docx]

**SUPPLEMENTAL MATERIALS AND GRAPHICS**

**Supplementary Tables**

**TableS1**: Screening of transposon mutants

| Strain | Description | Insertion site (amino acid residue) | Lesion length (mm) | EPS Production  (mg/ 10^9^ CFU) |
| --- | --- | --- | --- | --- |
| *Xcc* 8004 | Wild type | --- | 39.7± 10.6 | 1.34± 0.06 |
| *xdfA*B3 | Transposon mutant of *xdfA* | 17 | 9.3± 6.2 | 0.57± 0.17 |
| *xdfA*A2 | Transposon mutant of *xdfA* | 103 | 7.9± 2.7 | 0.53± 0.12 |
| *xdfA*P1 | Transposon mutant of *xdfA* | 132 | 13.8± 8.7 | 0.36± 0.07 |
| *xdfA*R6 | Transposon mutant of *xdfA* | 132 | 10.5± 3.4 | 0.58± 0.13 |
| ∆*xssA* | Virulence deficient mutant | --- | 6.5± 2.8 | --- |

**Table S2.** Strains and plasmids used in this study

| **Strains** | **Descriptions** | **Reference** |
| --- | --- | --- |
| *E. coli* K12 MG1655 | Wild type *Escherichia coli* | Lab collection |
| DH5α | F′/ endA1 hsdR17 (rk– mk+) supE44 thi-1 *recA1* *gyrA* *relA1* f80dlacZDM15 (*lacZYA-argF*) U169 | Lab collection |
| DH5/λ*pir* | Φ80*dlacZ*ΔM15 Δ(*lacZYAargF*) U169 *recA1 hsdR17 deoR thi-1 supE44 gyrA96 relA1*/λ*pir* | (1) |
| JW3597-1 | F-, *Δ(araD-araB)567*, *ΔlacZ4787*(::rrnB-3), *λ^-^*, *ΔrfaL734::kan*, *rph-1*, *Δ(rhaD-rhaB)568*, *hsdR514* | (2) |
| *Xcc* 8004 | Wild type *Xanthomonas campestris* pv. *campestris*; Rif^r^ (Rifampicin resistant) | Lab collection |
| *xdfAB3* | *xdfA*-17::pRL27; Kan^r^ (Kanamycin resistant) | This study |
| *xdfAA2* | *xdfA*-103::pRL27; Kan^r^ | This study |
| *xdfAR6* | *xdfA*-132::pRL27; Kan^r^ | This study |
| *xdfAP1* | *xdfA*-132::pRL27; Kan^r^ | This study |
| Δ*xdfA* | In frame deletion of *xdfA* (XC_2523); derivative of *Xcc* 8004; Rif^r^ | This study |
| Δ*xdfA*(XdfA^+^) | Chromosomal complementation (reconstitution) of *xdfA* (XC_2523); derivative of Δ*xdfA*; Rif^r^ | This study |
| Δ*xssA* | In frame deletion of *xssA* (XC_1107); derivative of *Xcc* 8004; Rif^r^ | (3) |
| *corA1* KO | Insertional knock out of *corA1* (XC_0628); derivative of *Xcc* 8004; Rif^r^, Kan^r^ | This study |
| Δ*corA2* | In frame deletion of *corA2* (XC_1781); derivative of *Xcc* 8004; Rif^r^ | This study |
| *corA1* KO/ Δ*xdfA* | Insertional knock out of *corA1* (XC_0628); derivative of Δ*xdfA*; Rif^r^, Kan^r^ | This study |
| Δ*corA2/* Δ*xdfA* | In frame deletion of *corA2* (XC_1781); derivative of Δ*xdfA*; Rif^r^ | This study |
| *corA1* KO/ Δ*corA2* | Insertional knock out of *corA1* (XC_0628); derivative of Δ*corA2*; Rif^r^, Kan^r^ | This study |
| *corA1* KO/ Δ*corA2/* Δ*xdfA* | Insertional knock out of *corA1* (XC_0628); derivative of Δ*corA2/* Δ*xdfA*; Rif^r^, Kan^r^ | This study |
| *Xcc* 8004 P*avrXccE*:*:gusA* | Chromosomal fusion *avrXccE* promoter with *gusA* using pVO155 in *Xcc* 8004 background, Rif^r^, Kan^r^, Ap^r^ | (4) |
| Δ*xdfA* P*avrXccE*:*:gusA* | Chromosomal fusion *avrXccE* promoter with *gusA* using pVO155 in Δ*xdfA* background, Rif^r^, Kan^r^, Ap^r^ | This study |
| *Xcc* 8004 P*hrpX*:*:gusA* | Chromosomal fusion *hrpX* promoter with *gusA* using pVO155 in *Xcc* 8004 background, Rif^r^, Kan^r^, Ap^r^ | (4) |
| Δ*xdfA* P*hrpX*:*:gusA* | Chromosomal fusion *hrpX* promoter with *gusA* using pVO155 in Δ*xdfA* background, Rif^r^, Kan^r^, Ap^r^ | This study |
| *Xcc* 8004 P*hrcU*:*:gusA* | Chromosomal fusion *hrcU* promoter with *gusA* using pVO155 in *Xcc* 8004 background, Rif^r^, Kan^r^, Ap^r^ | (4) |
| Δ*xdfA* P*hrcU*:*:gusA* | Chromosomal fusion *hrcU* promoter with *gusA* using pVO155 in Δ*xdfA* background, Rif^r^, Kan^r^, Ap^r^ | This study |
| **Plasmids** | **Descriptions** | **Reference** |
| pK18mobsacB | Km^r^ pUC18 derivative; lacZα mobs site sacB | (5) |
| pHM1 | Broad-host-range cosmid vector, pSa *ori*, Spec^r^ (Spectinomycin resistant) | (6) |
| pVO155 | pUC119 derivative carrying promoterless *gusA*; Kan^r^ Amp^r^ (Ampicillin resistant) | (7) |
| pRL27 | Tn5-RL27 (KmR-oriR6 K) delivery vector: circularized PCR fragment from pRL23 | (8) |
| pYP1 | pK18mobsacB with 420bp PCR fragment of *xdfA* 5’ end and a 321bp PCR fragment of *xdfA* 3’ end from the *Xcc* 8004 genome; Kan^r^ | This study |
| pYP2 | pK18mobsacB with 1338bp PCR fragment of *xdfA* full gene along with 5’ end and 3’ end from the *Xcc* 8004 genome; Kan^r^ | This study |
| pYP3 | pK18mobsacB with 1381bp PCR fragment of *xdfA* full gene having C-terminal HA tag along with 5’ end and 3’ end from the *Xcc* 8004 genome; Kan^r^ | This study |

**Table S3**. Oligonucleotides used in this study

| **Oligonucleotides** | **Sequence** |
| --- | --- |
| SCY_∆*xdfA_*F1_ EcoRI | GGGAATTCCGGGCGGATACAGCTTGAGCG |
| SCY_∆*xdfA_*R1_XbaI | GGTCTAGAAACGGATGAGCAGGGCGCAGA |
| SCY_∆*xdfA_*F2_XbaI | GGTCTAGATATCTTCATCGAGAAACTCTC |
| SCY_∆*xdfA_*R2_Hind III | GGAAGCTTCTCGAAGTGGGCACCGGCTCT |
| SCY_*xdfA* _HA_FO | TACCCATACGATGTTCCAGATTACGCTTGAGCAGGGCGCAGATGA |
| SCY_*xdfA* _HA_RO | AGCGTAATCTGGAACATCGTATGGGTATCCGTTCGTTGCCTTCCA |
| SCY_*xdfA* _FT_FO | GACTACAAAGACGATGACGACAAGTGAGCAGGGCGCAGATGA |
| SCY_*xdfA* _FT_RO | CTTGTCGTCATCGTCTTTGTAGTCTCCGTTCGTTGCCTTCCA |
| SCY_*xdfA* _OL_GFP_R1 | TTCTTCTCCTTTACTCATTCCGTTCGTTGCCTTCCACAC |
| SCY_*xdfA* _OL_GFP_F2 | GTGTGGAAGGCAACGAACGGAATGAGTAAAGGAGAAGAA |
| SCY_*xdfA* _OL_GFP_R2 | ATTTCATCTGCGCCCTGCTCATTTGTATAGTTCATCCAT |
| SCY_*xdfA* _OL_GFP_F3 | ATGGATGAACTATACAAATGAGCAGGGCGCAGATGAAAT |
| SCY_*xdfA_gusA*_FBamHi | CGGGATCCGGTGTCACCAGCTTCCAGCG |
| SCY_*xdfA_*GFP_REcoRI | GCTTAAGAAAACCCGGCCATGATACC |
| SCY_*xdfA_gusA*_RXbaI | GCTCTAGAAAAACCCGGCCATGATACC |
| SCY_*XCCcorA1*_Ins_F | CGGGATCCCTGGATGCAGTGGCCGCGCGG |
| SCY_*XCCcorA1*_Ins_R | ACGCGTCGACGGTCAGGTCCTTGTGCAGTAG |
| SCY_*XCCcorA2*_del_F1 | CCCAAGCTTAACCGTAATCTGCTGCTGGTC |
| SCY_*XCCcorA2*_del_R1 | GCTCTAGAGTTGTTCATGGCCGCATCGTG |
| SCY_*XCCcorA2*_del_F2 | GCTCTAGACTGTAAAGGGCCACCGTGTTG |
| SCY_*XCCcorA2*_del_R2 | GGAATTCGGCTACGCTCAGACAAGGGC |

**Table S4.** Amino acid sequence similarity of CorA1 (XC_0628) with its orthologs

| **RefSeq Accession number** | **Protein name (Organism name)** | **Amino acid identity** | **Amino acid similarity** |
| --- | --- | --- | --- |
| WP_004081315.1 | magnesium/cobalt transporter CorA [*Thermotoga maritima*] | 49/196(25%) | 90/196(45%) |
| AAA67612.1 | CorA [*Escherichia coli* str. K-12 substr. MG1655] | 78/314(25%) | 148/314(47%) |
| NP_253955.1 | magnesium transporter CorA family protein [*Pseudomonas aeruginosa*] | 91/298(31%) | 155/298(52%) |
| EAP73682.1 | magnesium/cobalt transporter CorA [*Ralstonia solanacearum*] | 76/298(26%) | 150/298(50%) |

**Table S5.** Amino acid sequence similarity of CorA2 (XC_1781) with its orthologs

| **RefSeq Accession number** | **Protein name [Organism name]** | **Amino acid identity** | **Amino acid similarity** |
| --- | --- | --- | --- |
| WP_004081315.1 | magnesium/cobalt transporter CorA [*Thermotoga maritima*] | 100/289(35%) | 162/289(56%) |
| AAA67612.1 | CorA [*Escherichia coli* str. K-12 substr. MG1655] | 43/152(28%) | 68/152(44%) |
| NP_253955.1 | magnesium transporter CorA family protein [*Pseudomonas aeureginosa*] | 74/304(24%) | 134/304(44%) |
| EAP73682.1 | magnesium/cobalt transporter CorA [*Ralstonia solanacearum*] | 70/309(23%) | 127/309(41%) |

**Supplementary Methods**

**Determination of sensitivity of the *Xcc* strains to various phenolics, detergents and osmolytes.**

The wild type *Xcc* along with Δ*xdfA* and Δ*xdfA* (XdfA^+^) were grown until late logarithmic phase. They were then normalized to an OD_600_ of 1.0 after which the cells were serially diluted in PBS pH 7.3. 2.5μl of these serially diluted cells were then spotted in triplicates onto PSA and additionally onto PSA medium supplemented with different phenolics like- Phenol, Acetosyringone, Berberine chloride, Rhein, and detergents like- sodium dodecyl sulfate, tween 20, triton X 100 (Sigma-Aldrich, St Louis). Similar experiment was performed on PSA plates supplemented with different osmolytes in a gradient of concentrations like- Mannitol, Sorbitol and Sucrose. The plates were then kept at 28°C for 48 hours and the difference in growth due to the presence of detergents was then compared with that of growth in PSA alone.

**LPS extraction of *Xcc* and *E. coli* strains**

The strains *Xcc* 8004, ∆*xdfA*, and ∆*xdfA* (XdfA^+^), as well as the *E. coli* strains *E. coli* (WT), and the LPS mutants JW3597, were grown to late log phase and normalized to an OD_600_ of 1.3. LPS was extracted from 1ml pellets of these OD_600_ normalized strains using the hot-aqueous phenol method, as described previously (9). DNase I and RNase were used to remove nucleotides from the samples. Additionally, the samples were also incubated for 3 hours with Proteinase K to remove protein contaminants. These samples were then heated at 65°C for 15 mins in Tris saturated phenol and the LPS were then extracted from the lower layer after centrifugation. 15µl of the extracted samples were then loaded onto 12% SDS- PAGE gel and visualized by silver staining.

**Construction of P*avrXccE*::*gusA*, P*hrpX*::*gusA*, P*hrcU*::*gusA*strains in *X. campestris* pv. *campestris* and ∆*xdfA* background**

Glucuronidase (GUS) reporter gene fusion was created by using the suicidal vector plasmid pVO155 having a promoter-less *gusA* gene(7). The respective putative promoter sequences of *avrXccE*, *hrpX* and *hrcU* gene clusters were amplified by using primers listed in Table S3 and integrated into pVO155 upstream of *gusA* gene. Subsequently, resulting constructs listed in Table S2 were introduced into *Xcc* wild-type and ∆*xdfA* by *E. coli* S17-1 mediated conjugation. Insertions of the GUS reporter cassette were confirmed by PCR using *gusA* specific and plasmid flanking primers.

***In planta* GUS expression assay**

In planta siderophore gene expression was studied by measuring β-glucuronidase activity. GUS marked *Xcc* strain and wild-type *Xcc* (control) were inoculated in the leaves of 45days old cabbage leaves. After 10 days of inoculation, leaves were crushed and dissolved in 1 ml of MUG extraction buffer (50mM sodium dihydrogen phosphate [pH 7.0], 10 mM EDTA, 0.1% sodium lauryl sarcosine, 0.1% Triton X-100, and 10 mM β-mercaptoethanol) without adding MUG substrate (4-methylumbelliferyl β-D-glucuronide). Subsequently, 250 μl extraction buffer containing MUG was added, and incubated at 37°C for appropriate time (10). Next, 75-μl aliquots were taken from each reaction mixture, and the reaction was terminated by the addition of 675 μl stop solution (0.2 M Na_2_CO_3_). Fluorescence was measured against 4-methyl-umbelliferone (MU; Sigma) as standard at excitation/emission wavelength of 365/455 nm, respectively. β-Glucuronidase activity for GUS was expressed as micromoles

of MU produced/minute/10^6^cells.

**Hypersensitivity response assay**

Four weeks old tomato S-22 cultivar and six weeks old *Nicotiana benthamiana* (acts as non-host for *Xanthomonas campestris* pv. *campestris*) were syringe-infiltrated with a suspension of *Xcc* strains and water control. Plants were incubated in green house for 24 h with minimum and maxium temperature of 26 and 28°C, respectively and relative humidity of 65%. Photographs of the leaves were captured at this stage to observe the browning of leaf due to hyper sensitivity (HR) response(11).

**Supplementary figure legends**

**Figure S1. Schematic representation of *xdfA* with its neighbourhoods.** Three independent Tn5 transposon insertion sites (represented by inverted triangles) are found at the corresponding amino acid residue 17, 103, and 132 in the *xdfA* locus (red coloured arrow). A length of 1 cm corresponds to 200 nucleotide bases in the schematic representation of the genomic organization in *Xcc* 8004 for the genes *xdfA* and its neighboring genes (blue colored arrow).

**Figure S2: Bacterial migration inside host cabbage plant.** Representative image of PSA plates showing *in planta* migration of *Xcc* strains, three days post inoculation.

**Figure S3: Growth phenotype under altered pH conditions.** Wild type *Xcc* 8004, ∆*xdfA* and ∆*xdfA*(XdfA^+^) were grown in rich PS broth in (A) pH 6.0, (B) pH 7.0, (C) pH 7.4, (D) pH 8.0, and (E) pH 9.0. The cultures were grown at 28°C and growth phenotypes are recorded as absorbance till 60^th^ hour post inoculation.

**Figure S4. Growth phenotype in presence of osmolytes.** The strains- *Xcc* 8004, ∆*xdfA* and ∆*xdfA*(XdfA^+^) were grown till mid log phase and normalized to OD_600_ 1.0, followed by serially dilution and spotting on PSA plates supplemented with 5%, 10% and 15% of Mannitol, Sorbitol and Sucrose. The experiment was performed in three biological replicates.

**Figure S5. Growth in presence of Phenolics and detergents.** Different Xcc strains were grown till mid log phase and normalised to OD_600_ 1.0 before serially diluted and spotted on PSA plates supplemented with 5mM Phenol, 1mM Acetosyringone, 10μg/mL Berberine chloride, 60μg/mL Rhein (top panel) 0.01% SDS, 0.2% Triton X 100 and 0.1% Tween 20 (bottom panel). The experiment was performed in three biological replicates.

**Figure S6.** Silver-stained Lipopolysaccharide profiling of the LPS mutant JW3596, JW3597, wild type *E. coli* along with *Xcc* 8004*,* ∆*xdfA* and ∆*xdfA*(XdfA^+^) on 12% SDS-PAGE. Boxes are drawn to indicate the differential banding pattern. These experiments were performed as three biological replicates.

**Figure S7. Hyper sensitivity response in nonhosts and transcriptional regulation of HR genes.** (A-B) *Nicotiana benthamiana* and *Lycopersicon esculentum* plants showing hypersensitive response 3 days post infiltration with different *Xcc* strains. (C) *in planta* GUS reporter assay with different *hrp* gene promoter expression pattern inside cabbage host. Error bars represent SD of the mean. *P* values were calculated using Student’s *t* test. (***p < 0.001).

**Figure S8. Elemental magnesium content measured by atomic absorption spectrometry (AAS).** (A) Intracellular magnesium content measured from the lyophilized cell pellet by AAS after growing the cells in rich PSA medium (B) Elemental magnesium content of the cell free supernatant and the media, measured by AAS after growing the cells for eight hours with or without supplementing 1mM MgSO_4_ to MM9 minimal media. All the experiments have been performed with three biological replicates.

**Figure S9.** **DedA is a ubiquitous protein group with no known structures or interactions.** Modified “sunburst” representation of the species tree for the DedA family of proteins (*SNARE_assoc* [PF09335]) generated using “pfam” domain of xfam.org. It is a simple graphical representation of the family across species. Each node in the tree is represented as a separate arc, arranged radially with the super kingdoms in the centre and the species arranged around the outermost ring.

**References:**

1. Miller VL, Mekalanos JJ. 1988. A novel suicide vector and its use in construction of insertion mutations: osmoregulation of outer membrane proteins and virulence determinants in Vibrio cholerae requires toxR. J Bacteriol 170:2575–2583.

2. Baba T, Ara T, Hasegawa M, Takai Y, Okumura Y, Baba M, Datsenko KA, Tomita M, Wanner BL, Mori H. 2006. Construction of Escherichia coli K-12 in-frame, single-gene knockout mutants: the Keio collection. Mol Syst Biol 2:2006.0008.

3. Pandey SS, Patnana PK, Rai R, Chatterjee S. 2017. Xanthoferrin, the α-hydroxycarboxylate-type siderophore of Xanthomonas campestris pv. campestris, is required for optimum virulence and growth inside cabbage. Molecular Plant Pathology 18:949–962.

4. Pandey SS, Patnana PK, Padhi Y, Chatterjee S. 2018. Low-iron conditions induces the hypersensitive reaction and pathogenicity hrp genes expression in Xanthomonas and is involved in modulation of hypersensitive response and virulence. Environmental Microbiology Reports 10:522–531.

5. Schäfer A, Tauch A, Jäger W, Kalinowski J, Thierbach G, Pühler A. 1994. Small mobilizable multi-purpose cloning vectors derived from the Escherichia coli plasmids pK18 and pK19: selection of defined deletions in the chromosome of Corynebacterium glutamicum. Gene 145:69–73.

6. Innes RW, Hirose MA, Kuempel PL. 1988. Induction of nitrogen-fixing nodules on clover requires only 32 kilobase pairs of DNA from the Rhizobium trifolii symbiosis plasmid. J Bacteriol 170:3793–3802.

7. Oke V, Long SR. 1999. Bacterial genes induced within the nodule during the Rhizobium–legume symbiosis. Molecular Microbiology 32:837–849.

8. Larsen RA, Wilson MM, Guss AM, Metcalf WW. 2002. Genetic analysis of pigment biosynthesis in Xanthobacter autotrophicus Py2 using a new, highly efficient transposon mutagenesis system that is functional in a wide variety of bacteria. Arch Microbiol 178:193–201.

9. Michael R. Davis J, Goldberg JB. 2012. Purification and Visualization of Lipopolysaccharide from Gram-negative Bacteria by Hot Aqueous-phenol Extraction. JoVE (Journal of Visualized Experiments) e3916.

10. Jefferson RA, Kavanagh TA, Bevan MW. 1987. GUS fusions: beta-glucuronidase as a sensitive and versatile gene fusion marker in higher plants. The EMBO Journal 6:3901–3907.

11. Rai R, Ranjan M, Pradhan BB, Chatterjee S. 2012. Atypical Regulation of Virulence-Associated Functions by a Diffusible Signal Factor in Xanthomonas oryzae pv. oryzae. MPMI 25:789–801.
